# Early Evolution of the Mitogen-Activated Protein Kinase Family in the Plant Kingdom

**DOI:** 10.1038/s41598-019-40751-y

**Published:** 2019-03-11

**Authors:** Balázs Kalapos, Monika Hlavová, Tímea V. Nádai, Gábor Galiba, Kateřina Bišová, Róbert Dóczi

**Affiliations:** 1 Institute of Agriculture, Centre for Agricultural Research of the Hungarian Academy of Sciences, H-2462 Martonvásár, Brunszvik u. 2, Hungary; 2 Festetics Doctoral School, Georgikon Faculty, University of Pannonia, 8360 Keszthely, Hungary; 3 Centre Algatech, Institute of Microbiology Academy of Sciences of the Czech Republic, Opatovicky mlyn, CZ 379 81, Třeboň, Czech Republic

## Abstract

Mitogen-activated protein kinase (MAPK) pathways are central cellular signalling mechanisms in all eukaryotes. They are key regulators of the cell cycle and stress responses, yet evolution of MAPK families took markedly different paths in the animal and plant kingdoms. Instead of the characteristic divergence of MAPK types in animals, in plants an expanded network of ERK-like MAPKs has emerged. To gain insight into the early evolution of the plant MAPK family we identified and analysed MAPKs in 13 representative species across green algae, a large and diverse early-diverging lineage within the plant kingdom. Our results reveal that the plant MAPK gene family emerged from three types of progenitor kinases, which are ubiquitously present in algae, implying their formation in an early ancestor. Low number of MAPKs is characteristic across algae, the few losses or duplications are associated with genome complexity rather than habitat ecology, despite the importance of MAPKs in environmental signalling in flowering plants. ERK-type MAPKs are associated with cell cycle regulation in opisthokont models, yet in plants their stress-signalling function is more prevalent. Unicellular microalgae offer an excellent experimental system to study the cell cycle, and MAPK gene expression profiles show CDKB-like peaks around S/M phase in synchronised *Chlamydomonas reinhardtii* cultures, suggesting their participation in cell cycle regulation, in line with the notion that the ancestral eukaryotic MAPK was a cell cycle regulator ERK-like kinase. Our work also highlights the scarcity of signalling knowledge in microalgae, in spite of their enormous ecological impact and emerging economic importance.

## Introduction

The mitogen-activated protein kinase (MAPK) pathways are pivotal regulatory mechanisms in all eukaryotes^1^. The canonical MAPK pathway consists of three types of enzymes (MAPK, MAPK kinase [MKK] and MAPK kinase kinase [MAPKKK]), which constitute signalling pathways where signal transmission operates via an activating phosphorylation cascade.

Activation of MAPKs occurs by the dual phosphorylation of a TxY motif in the activation loop sequence by upstream MKKs. In yeast and mammals the central amino acid of the TxY phosphorylation motif is characteristic of the major MAPK pathways. The KSS1 pathway of yeast (activated by mating pheromones) and the ERK pathway of mammals (activated by growth factors and mitogens) constitute of MAPKs with TEY phosphorylation motifs. MAPKs of the yeast HOG1 pathway (activated by osmotic stress) and the mammalian p38 pathway (activated by inflammatory cytokines and stress) possess TGY motifs and the mammalian stress-activated JNKs have TPY motifs^2,3^. Quite contrastingly, MAPKs in plants have not diverged into such distinct activation loop variants, the central residue is either glutamic acid (TEY) or the similar aspartic acid (TDY). Plant MAPKs are most related to ERK-type animal MAPKs^4^, and it has been proposed that the ancestral eukaryotic MAPK was an ERK-like kinase^5^. During the course of land plant evolution the T[E/D]Y family has largely expanded, giving rise to MAPK families in flowering plants (angiosperms) that outnumber mammalian MAPKs. Connectivity within flowering plant MAPKs and MKKs is remarkably high, indicating the lack of separate pathways and the existence of a highly complex network^5,6^.

Interestingly, ERK-type MAPK pathways in yeast and animals mainly function in cell cycle regulatory roles and thus many cancer models are associated with mutations of the ERK pathway, whereas stress signalling is predominantly associated with other pathways, such as p38 and JNK. Yet, although the role of plant MAPKs in the cell cycle (mainly in cytokinesis) is reported in several publications, and various developmental processes are evidently regulated by them^7^, the central role of T[E/D]Y MAPKs in stress signalling is supported by a far more substantial amount of evidence^8–12^. Thus, current knowledge clearly underscores different evolutionary paths for the MAPK families in the two major eukaryotic clades, however, early events that defined the emergence of the plant-specific branch of the MAPK family are largely unclear.

Photosynthetic green microalgae (Chlorophyta) are a large and diverse, early diverging group within the plant kingdom (Viridiplantae), consisting of uni- and multi-cellular species, which are distributed across a wide range of physiological and ecological conditions. Comparative analysis of gene families across microalgae and more complex land plants can facilitate reconstruction of the gene family’s evolutionary history prior to separation from the last common ancestor. Besides, the importance of understanding adaptation mechanisms in microalgae is also underscored by the significance of this group. For example, microalgae have a tremendous ecological impact, e.g. they are a major producer of atmospheric oxygen^13^ and are a main component at the base of the aquatic food chain. Recently the rapid rise of marine planktonic algae during the transition between Cryogenian and Ediacaran periods (659–645 million years ago) has been proposed as the main driver of the evolution of increasingly complex organisms, such as multicellular animals^14^. Besides ecological importance, algal biotechnology has become a rapidly developing field for producing nutrients, pharmacological substances or cosmetics, exploiting the rich repertoire of algal secondary metabolites as high value products. Algae are also envisaged as the ideal producers of next generation biofuels^15,16^. At this point the use of algae for biofuels is hindered by high costs of the biomass production. Novel approaches such as usage of wastewater instead of fertilisers, and most importantly usage of waste CO_2_ for supporting algal growth bring the double benefits of decreasing the price of algal biomass and bioremediating waste water and CO_2_.

In experimental practice the unicellular green alga, *Chlamydomonas reinhardtii* has become an important model organism due to its sequenced genome^17^, suitability for transformation, the accessibility of well-characterised strains, vectors and protocols (http://chlamy.org/) and the availability of an indexed mutant collection^18^. Due to its unicellular nature, *Chlamydomonas* is an excellent model system for plant-related cellular biological processes and has been utilised to study photosynthesis, lipid biosynthesis or the cell cycle. Yet, despite the ubiquitous importance of microalgae, the knowledge on their physiological adaptation mechanisms and in particular on the signal transduction mechanisms that regulate the underlying molecular responses is very limited. Importantly, physiological knowledge obtained from photosynthetic microalgae has implications for the evolution of these processes in the plant kingdom.

Therefore, in order to gain insight into the early evolutionary history of the plant MAPK gene family, we set out to analyse the proteomes of 13 diverse species of green algae for a detailed phylogenetic analysis of algal MAPKs and also asked if algal MAPKs are associated with the cell cycle by assaying their expression profiles throughout the cell cycle in the algal model species, *C. reinhardtii*.

## Results

### Identification of MAPK Families in Algal Proteomes

**Figure 1:**
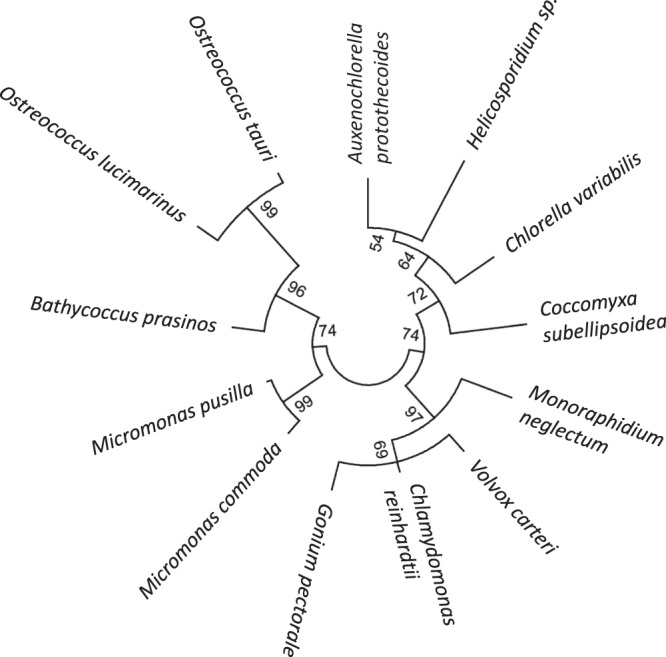
Phylogenetic relationships of the 13 algal species used in this study. Actin protein sequences of the species described in Table 1 were aligned by the MUSCLE alignment method and the phylogenetic tree was constructed by the Maximum-likelihood method using the MEGA6 software package. The tree represents the known phylogenetic relationships of the selected species, separation of the three major classes (Prasinophyceae, Trebouxiophyceae and Chlorophyceae) is evident. There is a distinct split within the marine picoeukaryotes of Prasinophyceae, where the two *Ostreococcus* species and *B. prasinos* form a separate clade.

In order to gain a comprehensive inventory of algal MAPKs we screened 13 algal species with sequenced genomes to identify MAPK sequences. The 13 species represent a wide range of green algae in terms of phylogeny, life style, geographical distribution, ecological or economic importance. For example, they represent the three major classes, including widely distributed cosmopolitan marine planktonic or fresh water species as well as highly specialised species adapted to extreme conditions or parasitic life style. The species also represent the spectrum of cell sizes, genome sizes and gene numbers across green algae. A detailed summary of the species included in this study is presented in Table 1. The phylogenetic relationships of the 13 species were assessed by generating a phylogenetic tree based on actin protein sequences (Fig. 1).

**Table 1:** Green algal species in this study.

Species and descriptions	Classification Class Order Family	genome size (Mbp)	no. of genes	GC content (%)
***Bathycoccus prasinos*** Extremely small, cosmopolitan marine species, cells are covered with scales. An important component of the picoeukaryote compartment. Sequenced strain isolated from surface water^41^.	Prasinophyceae Mamiellales Prasinophyceae	15	7847	48
***Ostreococcus lucimarinus CCE9901*** ***Ostreococcus tauri*** Cosmopolitan marine primary producers. The smallest known eukaryotes (~1-μm diameter). Extremely simple cellular organization: no cell wall and flagella, a single chloroplast and mitochondrion^42,43^.	Prasinophyceae Mamiellales Prasinophyceae	13.2 12.6	7651 8116	60 59
***Micromonas commoda RCC299*** ***Micromonas pusilla CCMP1545*** Cosmopolitan marine picoeukaryotes (<2 µm). Motile, with a single chloroplast and mitochondrion^44^.	Prasinophyceae Mamiellales Mamiellaceae	20.9 21.9	10056 10575	64 65
***Auxenochlorella protothecoides*** Excellent candidate for commercial manufacture of microalgae-derived biofuel. Capable for both autotrophism (photosynthesis) and heterotrophism (glucose fermentation). Upon switching to heterotrophism the chloroplast is replaced by lipid bodies, leading to high oil accumulation^45^.	Trebouxiophyceae Chlorellales Chlorellaceae	22.9	7039	63
***Chlorella variabilis*** Chlorella sp. are small (~2 to 10 μm in diameter), unicellular, coccoid, nonmotile and contain a single chloroplast. Highly interesting for biotechnological applications, e.g. biofuels, sequestering CO_2_, producing molecules of high economic value, removing heavy metals from wastewaters. Model system for virus/algal interactions. The sequenced strain is a hereditary photosynthetic endosymbiont of the unicellular protozoan *Paramecium bursaria*^46^.	Trebouxiophyceae Chlorellales Chlorellaceae	46	9791	67
***Helicosporidium sp. ATCC 50920*** A gut parasite of the black fly *Simulium jonesi*^47^.	Trebouxiophyceae Chlorellales Chlorellaceae	17	6035	62
***Coccomyxa subellipsoidea C-169*** Small (3 to 9 μm) elongated non-motile unicellular green alga isolated in Antarctica, tolerant to extreme cold^48^.	Trebouxiophyceae unclassified Coccomyxaceae	48.8	9851	53
***Chlamydomonas reinhardtii*** Unicellular green alga (10 µm), motile with two flagella. Able to grow photoautotrophically and heterotrophically. Considered as the model algal species, extensively used to study photosynthesis, lipid biosynthesis and the cell cycle. Commercially interesting for producing biopharmaceuticals and biofuels^17^.	Chlorophyceae Chlamydomonadales Chlamydomonadaceae	121	15143	64
***Monoraphidium neglectum*** The sequenced strain is a robust biomass accumulator, and produces high lipid contents under nitrogen starvation. Has a very good potential for biofuel production^49^.	Chlorophyceae Sphaeropleales Selenastraceae	68	16761	65
***Gonium pectorale*** An undifferentiated multicellular species. Used to study the initial steps in the evolution of multicellularity^50^.	Chlorophyceae Volvocales Goniaceae	149	17984	65
***Volvox carteri f. nagariensis*** A differentiated multicellular species, with two cell types: small biflagellate somatic and large reproductive cells. Used to study the evolution of multicellularity and development^51^.	Chlorophyceae Volvocales Chlorophyceae	138	14520	56

To identify MAPK sequences we performed a Hidden Markov Model (HMM) based screen of the 13 proteomes, which identified 39 canonical MAPK proteins (Table 2). Atypical MAPKs^19^ were excluded from further analysis because these kinases do not participate in conventional MAPK cascades and their function in plants is obscure. One exception is the closely related ERK8-like group, which has an intriguing evolutionary history in plants. ERK8 emerged in a very early ancestral eukaryote, as ERK8-like kinases are found in protists, animals and lower plants, including green algae, but they are lost in flowering plants^5^. Therefore we opted to include ERK8-like sequences in our analyses of algal MAPKs (Table 2).

**Table 2:** MAPK proteins identified in the 13 species of algae.

Species	Protein ID	Protein Size (aa)	Conserved Phosphorylation Site Motif (domain VIII)	Phylogenetic Group
*Bathycoccus prasinos*	CCO18034	535	FMTEYVVTRWYRAPELLLSC	A/B
	CCO16213	535	FWTDYVATRWYRAPELCGSF	D
*Ostreococcus lucimarinus*	XP_001419851	405	FMTEYVVTRWYRAPELLLSC	A/B
	XP_001419478	449	MMTSYVVTRWYRAPELLLNS	C
	XP_001419059	387	FWTDYVATRWYRAPELCGSF	D
*Ostreococcus tauri*	XP_003081390	459	FMTEYVVTRWYRAPELLLSC	A/B
	XP_003083155	424	MMTSYVVTRWYRAPELLLNS	C
	XP_003080614	507	FWTDYVATRWYRAPELCGSF	D
*Micromonas commoda*	XP_002503699	415	FMTEYVVTRWYRAPELLLSC	A/B
	XP_002501054	360	FMTEYVVTRWYRAPELLLSC	C
	XP_002503335	370	FWTDYVATRWYRAPELCGSF	D
	XP_002507666	363	ILTDYVATRWYRAPEILLGS	ERK8-like
*Micromonas pusilla*	XP_003059282	426	FLTEYVVTRWYRAPELLLSC	A/B
	XP_003057263	374	FMTEYVVTRWYRAPELLLSC	C
	XP_003058405	362	FWTDYVATRWYRAPELCGSF	D
	XP_003056520	380	VLTDYVATRWYRAPEILLGS	ERK8-like
*Auxenochlorella protothecoides*	XP_011398170	384	FMTEYVVTRWYRAPELLLSC	C
	XP_011396366	257	VLTDYVATRWYRAPEILLSS	ERK8-like
	XP_011399372	469	FWTDYVATRWYRAPELCGSF	D
*Chlorella variabilis*	XP_005848327	360	FMTEYVVTRWYRAPELLLSC	C
	XP_005844785	566	FWTDYVATRWYRAPELCGSF	D
	XP_005846540	409	VLTDYVATRWYRAPEILLSS	ERK8-like
*Helicosporidium* sp. ATCC 50920	KDD75646	375	LMTEYVVTRWYRAPELLLSC	A/B
	KDD76079	270	FMTEYVVTRWYRAPELLLSC	C
	KDD74687	461	FWTDYVATRWYRAPELCGSF	D
	KDD76312	386	VLTDYVATRWYRAPEILLSS	ERK8-like
*Chlamydomonas reinhardtii*	XP_001700291	389	FMTEYVVTRWYRAPELLLSC	A/B
	XP_001690850	375	FMTEYVVTRWYRAPELLLSC	C
	XP_001690852	383	FMTEYVVTRWYRAPELLLSC	C
	XP_001694300	398	FWTDYVATRWYRAPELCGSF	D
	XP_001703601	353	LWTDYVATRWYRAPELCGCF	D
	XP_001699095	353	ILTDYVATRWYRAPEILLGS	ERK8-like
*Coccomyxa subellipsoidea*	XP_005651434	377	YMTEYVVTRWYRAPELLLSC	C
	XP_005645479	537	FWTDYVATRWYRAPELCGSF	D
	XP_005646693	391	ILTDYVATRWYRAPEILLGS	ERK8-like
*Monoraphidium neglectum*	XP_013902829	343	MLTEYVVTRWYRAPELLLSC	A/B
	XP_013893971	314	FMTEYVVTRWYRAPELLLSC	C
	XP_013900021	697	FWTDYVATRWYRAPELCGSF	D
*Gonium pectorale*	KXZ46360	394	FMTEYVVTRWYRAPELLLSC	A/B
	KXZ55952	388	FMTEYVVTRWYRAPELLLSC	C
	KXZ56454	691	MWTDYVATRWYRAPELCGCF	D
	KXZ43541	642	FWTDYVATRWYRAPELCGSF	D
	KXZ50454	442	ILTDYVATRWYRAPEILLGS	ERK8-like
*Volvox carteri*	XP_002955338	397	FMTEYVVTRWYRAPELLLSC	A/B
	XP_002948304	381	FMTEYVVTRWYRAPELLLSC	C
	XP_002954138	346	LWTDYVATRWYRAPELCGCF	D
	XP_002954367	466	FWTDYVATRWYRAPELCGSF	D
	XP_002951120	336	ILTDYVATRWYRAPEILLGS	ERK8-like

The number of MAPKs in algal genomes is relatively even, most species (6 of 13) contain three MAPKs. Four species have only two, while two and one species with four and five MAPKs, respectively, constitute the most MAPK-rich species (Table 2), indicating possible linage-specific gene losses and duplications, respectively. Gene losses are associated with more compacted genomes. Nevertheless, there are species with three MAPK types in all major clades, strongly implying that lack of certain MAPKs is a consequence of gene loss. Both TEY and TDY phosphorylation motifs occur in all species, supporting a very early divergence of these two MAPK types. Besides the set of canonical MAPKs most species contain a single ERK8-like sequence, although this kinase type is apparently lost in four species. Interestingly, the ERK8-like kinases possess TDY-type phosphorylation motifs throughout algae, whereas this motif is TEY in human ERK8.

**Figure 2:**
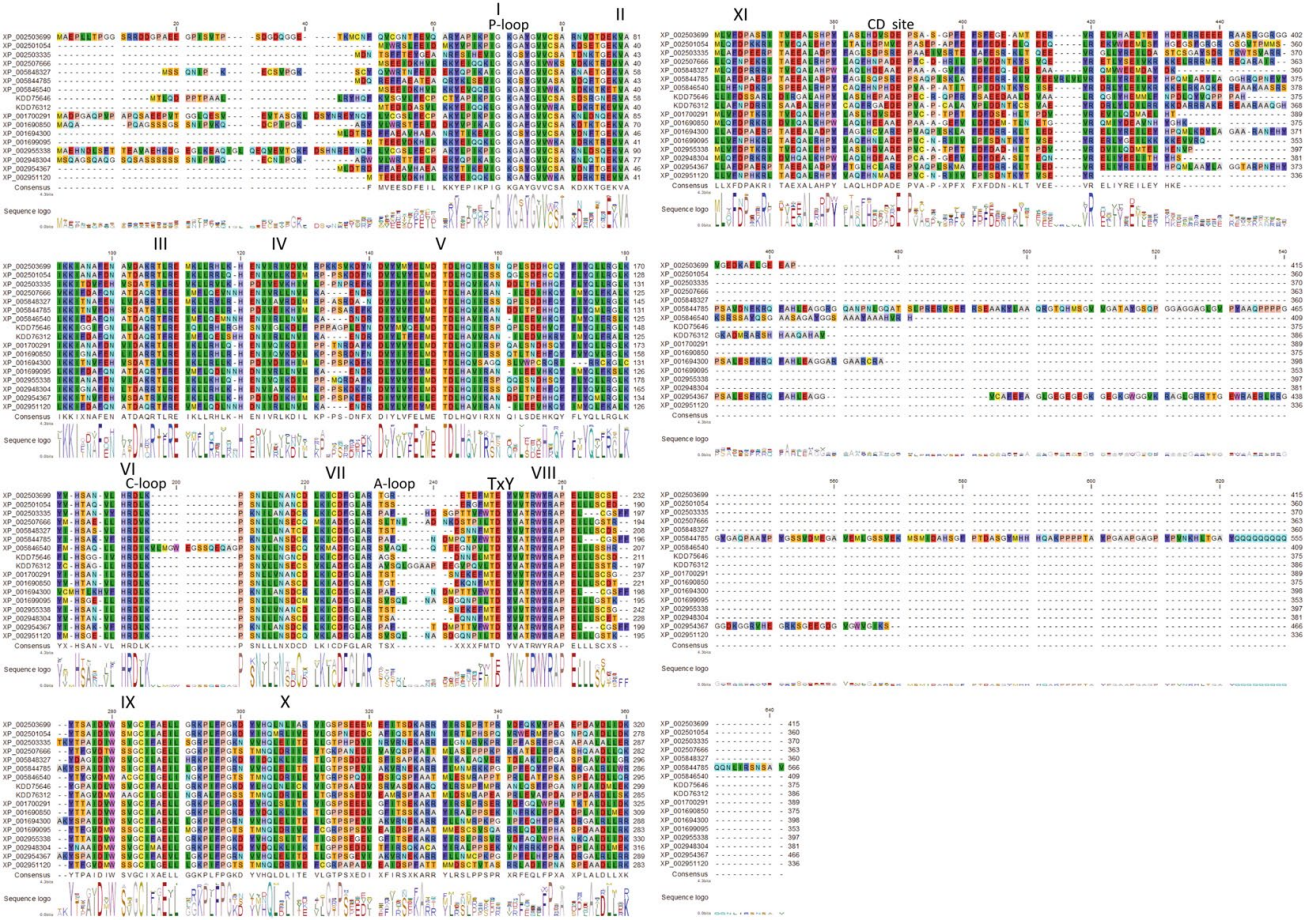
Multiple alignment of selected algal MAPK protein sequences. 17 representative sequences of various species and phylogenetic groups were aligned by the MUSCLE alignment method. Amino acids are coloured according to their biochemical properties to visualise conservation and similarities. Consensus is indicated below each block. The 11 conserved protein kinase subdomains are highlighted using Roman numerals (I to XI) above the sequences. The characteristic kinase motifs, P-loop, C-loop and A-loop are indicated above the alignments. The typical MAPK-specific phosphorylation motif (TxY) and the CD site are also indicated above the alignments. Alignment of the entire set of MAPK sequences in this study is available in Supplementary Fig. S1.

**Figure 3:**
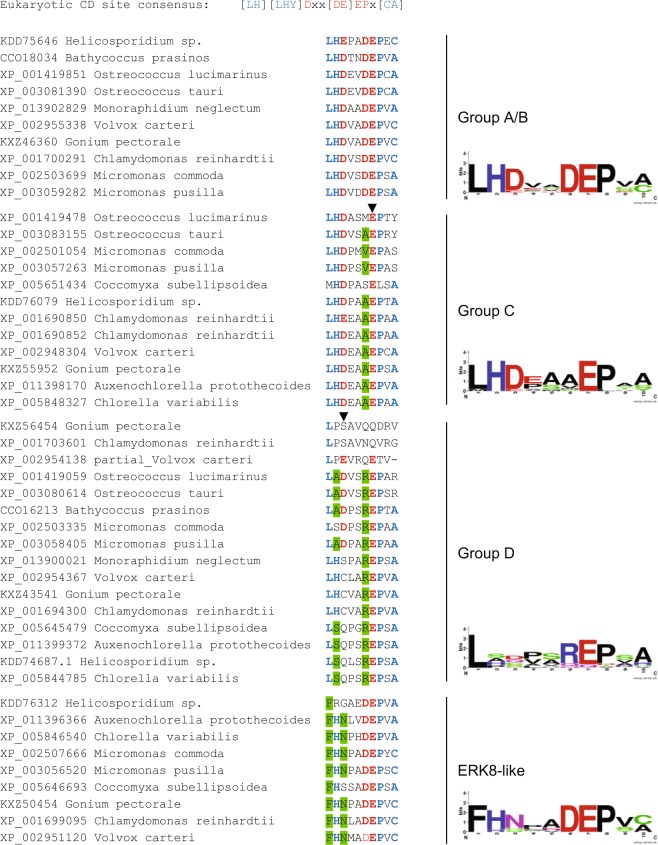
Docking site sequences and sequence logos by phylogenetic groups. The conserved eukaryotic MAPK common docking (CD) motif is indicated above, highly conserved residues are indicated by blue font, acidic amino acids are indicated by red font, for better visualisation. The CD site regions of all algal MAPKs are shown on the left, grouped according to phylogenetic groups, as indicated on the right. Group-specific systematic amino acid substitutions are highlighted by green. Arrowheads indicate positions where further systematic changes took place in higher plants: in group C, an E/N substitution (position 7), in group D, the appearance of a K (position 3), bringing about a second charge swap on top of the R in position 6^5^. These substitutions suggest that algal MAPKs represent intermediate forms of drifting group-specific CD site evolution. Sequence logos of the CD site patterns by groups were generated by WebLogo^52^, and are shown on the right.

Sequence alignment indicated the presence of the 11 highly conserved subdomains that constitute protein kinases (Fig. 2 and Supplementary Fig. 1). Characteristic features of MAP kinases, such as the phosphate-binding loop (P-loop), activation-loop (A-loop), and catalytic loop (C-loop), are also evident. The common docking (CD) site, which mediates interaction with D-site containing proteins (i.e. activating MKKs and substrates) and is conserved throughout eukaryotes^20^, is present in its complete form only in one phylogenetic group (A/B). In group C one conserved acidic residue is lost, while further positions are substituted in group D and the ERK8-like kinases (Fig. 3).

### Phylogenetic Analysis Reveals Early Formation of Three Basic Plant MAPK Types

**Figure 4:**
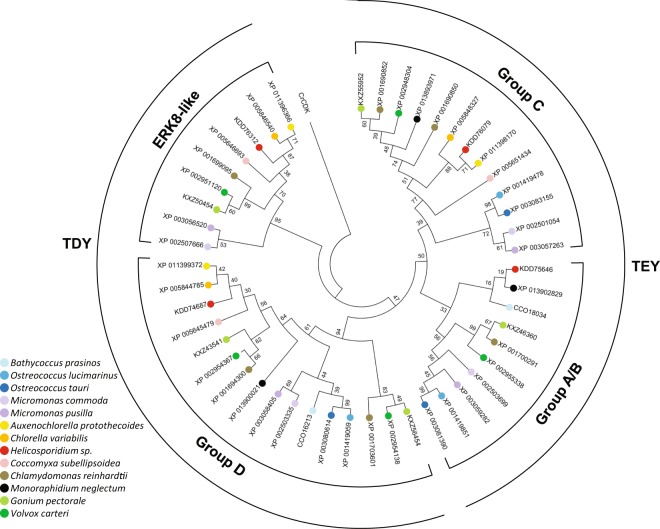
Phylogenetic relationships of MAPK sequences in 13 algal species. Amino acid sequences of the kinase domains of the 48 identified MAPKs (Table 2) were aligned by the MUSCLE alignment method and the phylogenetic tree was constructed by the Maximum-likelihood method using the MEGA6 software package. A cyclin-dependent kinase (CDKG1) of *Chlamydomonas reinhardtii* was used as outgroup. Species are indicated by each protein ID in the tree with coloured dots according to the key on the left. Phylogenetic grouping and the type of the conserved phosphorylation site (TxY) are indicated at the inner and outer perimeters, respectively. Phylogenetic trees generated by using full-length amino acid and nucleotide sequences are provided in Supplementary. Figs S2, S3, respectively.

Phylogenetic classification of flowering plant MAPKs is well established. TEY-type kinases form three groups, designated A-C, while the TDY clade is designated group D^21^. It was proposed that group C and group D type MAPKs can also be found in algal species, while groups A and B separated in land plants. An intermediate clade between C and land plant A and B groups was designated A/B, which may represent the progenitor kinase type of land-plant group A and B MAPKs^5^. For a reliable reconstruction of the early evolutionary history of green MAPKs we performed phylogenetic analyses using three sequence types: full-length amino acid, kinase domain amino acid and CDS nucleotide sequences. Although the mutation rates of the three sequence types are different (due to factors such as silent mutations or purifying evolution of the kinase domains) the resulting trees are very similar in architecture, indicating a credible reconstruction of evolutionary relationships (Fig. 4 and Supplementary Figs S2, S3).

In all trees three major clades formed. The ERK8-like clade is well separated from the canonical MAPKs, in agreement with the early separation of this kinase type, prior to the separation of the plant lineage. Within the canonical MAPKs TEY and TDY type sequences form two major clades, while the TEY clade is further separated into two major clades, formed by members of groups C and A/B. Interestingly, group C MAPKs of the tiny planktonic Prasinophyceae form a separate small subclade in the amino-acid-based trees. In the nucleotide tree Prasinophyceae TEY sequences fully separate from other species, in accordance with the higher mutation rate at the nucleotide level. Divergence of the Prasinophyceae TEY sequences is in line with the larger evolutionary distance of this group.

### Evidence of Local Release of Purifying Selection That May Contribute to Functional Divergence Following Duplication of MAPKs

**Figure 5:**
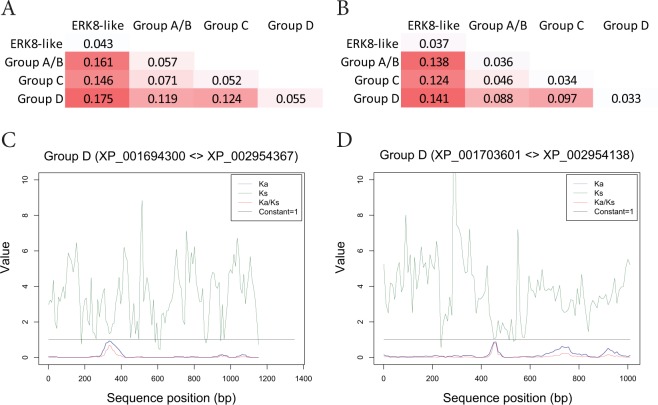
Predominance of purifying selection with evidence for localised release following gene duplication during the evolution of plant MAPKs. (**A**,**B**) Ka/Ks values were calculated for all pairwise combinations of the 48 algal MAPKs (Supplementary. Tables S1, S2). For visualisation, the average values of all pairwise combinations within each phylogenetic group combinations are presented for full-length sequences (**A**) and kinase domains. (**B**) Ka/Ks values below 1.0 indicate purifying selection. (**C**,**D**) Sliding window analysis of sequence divergence between two group D MAPKs in *C. reinhardtii* and *V. carteri*, representing a lineage-specific gene duplication event. For sliding window analysis, the full-length nucleotide sequences of the indicated orthologous MAPKs were used at 51-bp window size and 9-bp step size settings. The Ka/Ks plots are indicated by red lines.

Our results reveal the existence of three basic MAPK types across algae, strongly implying that the canonical plant MAPK family evolved from three MAPK forms in an early common plant ancestor. In order to gain insight into the selection forces acting on diverging MAPKs we calculated Ka/Ks ratios across the identified sequences (Fig. 5, Supplementary Fig. S4 and Tables S1, S2). In line with the high degree of conservation of MAPKs, which mainly consist of a kinase domain sequence, the overall Ka/Ks values are low, indicating strong purifying selection. Ka/Ks ratios are higher between phylogenetically more distant groups than between more closely related groups or especially within groups. To better visualise this trend, we calculated the average Ka/Ks values for each pairwise group relation (Fig. 5A,B). While the average Ka/Ks values within each group are very similar, they increase in proportion to the phylogenetic distance between groups, indicating that neofunctionalisation of the diverging MAPK types takes place, despite strong negative selection. Purifying selection serves the protection of kinase catalytic activity, and accordingly the Ka/Ks ratios are lower when calculated for the kinase domains only (Fig. 5A,B).

For a more refined assessment of the selection forces driving sequence variation we performed a sliding window analysis to identify potential regions or sites under less stringent purifying selection. To this end MAPK orthologues of the relatively closely related Chlorophyceae species *C. reinhardtii* and *V. carteri* were used, where a lineage-specific duplication of group D MAPKs occurred. The Ka/Ks values are very low throughout the most part of all sequences, however in A/B (Cr/VcMPK8) and C (Cr/VcMPK3) type MAPK orthologues the Ka/Ks ratios are increased in the N-terminal regions outside of the kinase domains, indicating possible sites of functional divergence (Supplementary Fig. 6). Remarkably, in the two paralogous group D MAPKs one region of significantly increased Ka/Ks ratios was detected within the kinase domain: in case of Cr/VcMPK2 between the conserved subdomains V and VI, while in case of Cr/VcMPK4 between subdomains VII and VIII (Fig. 5C,D). These findings imply accelerated neofunctionalisation following duplication of a MAPK gene through locally released negative selection, which may even be located within the otherwise highly conserved kinase domain.

### Gene Expression Profiles of MAPK Genes Suggest Functional Relevance in Cell Cycle Regulation

In opisthokont species ERK-type MAPKs are involved in cell cycle regulation, and thus mutations affecting the ERK pathway are highly associated with cancers. A previous transcriptome analysis of synchronized *C. reinhardtii* cultures suggested cell cycle specific expression of MAPK-encoding genes^22^. To investigate transcriptional regulation of MAPKs during the cell cycle, we synchronized phototrophic cultures by a 13 h light/11 h dark regime, sampled the cultures hourly and analysed gene expression by quantitative RT-PCR. The experiment was performed three times with similar results. During the cultivation period, the cells increased their size by 10-fold and divided into 4, 8 or rarely 16 daughter cells. At the 4^th^ hour of the cell cycle, all cells have attained the first commitment point (CP) for division; three to four rounds of protoplast divisions were completed by the 13^th^ hour of the cell cycle. In comparison to the cultures used by Zones and colleagues^22^ our cultures had shorter cell cycle and divided earlier although the number of daughter cells was comparable.

We analysed the expression profile of the plant-specific cyclin-dependent kinase, *CDKB*, which is specifically expressed during alternating S phase(s) and mitosis(es) (S/M phase) just prior to protoplast division and served as a control, and six MAP kinases (*MPK2*, *MPK3*, *MPK4*, *MPK5*, *MPK6* and *MPK8*). Since in our hands many widely used reference genes such as actin, tubulin, ubiquitin, or G-protein β-subunit-like protein (GBLP) show a cell cycle regulated expression we normalized the gene expression data by the NORMA-Gene software^23^, which is able to overcome such limitations. *CDKB* expression showed two characteristic peaks. The first one occurred at about the 3^rd^ hour of the cell cycle and corresponded to the passage through commitment point. The second peak with a maximum around the 10^th^ hour of the cell cycle corresponded to S/M phase. This expression profile is consistent with previously published qRT-PCR results^24^ and the peak at S/M phase is consistent with transcriptomics data^22,25^.

**Figure 6:**
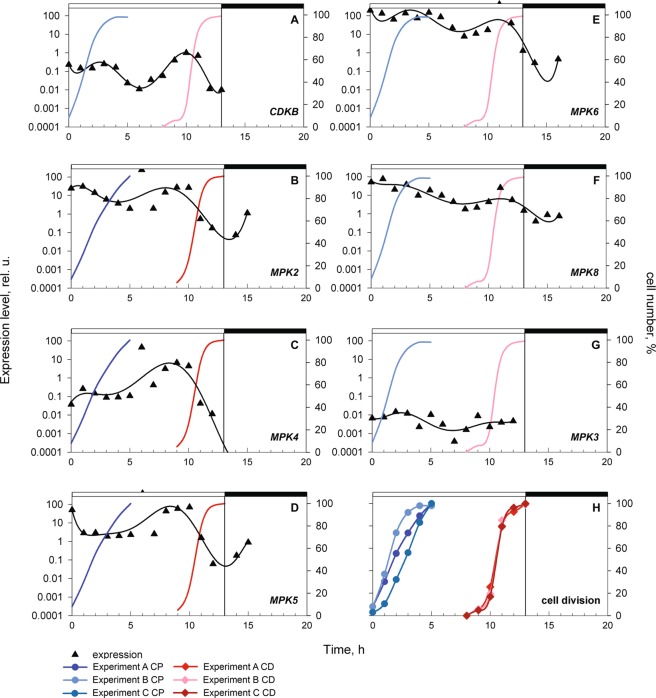
Cell cycle progression of synchronized population of wild type culture *Chlamydomonas reinhardtii* (**H**) and expression profiling of CDKB (**A**), MPK2 (XP_001694300) (**B**), MPK4 (XP_001703601) (**C**), MPK5 (XP_001699095) (**D**), MPK6 (XP_001690850) (**E**), MPK8 (XP_001700291) (**F**) and MPK3 (XP_001690852). (**G**) The graph (**H**) shows the percentages of cells that attained first commitment point (CP) (blue curves, filled circles) and completed cell division (dark red curves, filled diamonds) in three biological replicates. At the 4^th^ hour of the cell cycle, all cells have attained the first CP; cell divisions were completed by the 13^th^ hour of the cell cycle. The changes in transcript levels of CDKB (**A**) and MPK genes (**B**–**G**) are depicted by black curves (triangles). Gene expression was analysed by qRT-PCR, the resulting Ct values were treated by NORMA-Gene macro to get a normalized expression of each gene. For presentation, the data was normalized against the highest value of the CDKB gene expression. The attainment of CP and progression through cell division is depicted by blue or red curves in each graph. Light and dark periods are indicated above the panel by empty and filled vertical bars, respectively.

Our results revealed that the expression of most *MAPK* genes peaks at the beginning of S/M phase followed by a drop in expression (Fig. 6), in a manner similar to the main peak of *CDKB*, although with varying peak intensities. The most similar peaking pattern to *CDKB* was detected in case of *MPK4* and *MPK5*, while *MPK2*, *MPK6* and *MPK8* displayed a moderate increase of expression around of S/M phase, followed by a drop in expression. Expression of *MPK3* was several orders of magnitude lower than the other tested *MAPK* genes, and *MPK3* transcript was constitutively present during the cell cycle.

The expression patterns of *MPK2*, *MPK3*, *MPK4* and *MPK5* are consistent with the results obtained by RNA-seq transcriptomics^22^, while more pronounced expression peaks at S/M phase were detected for *MPK6* and *MPK8* by Zones and colleagues. These results highlight a specific, S/M phase-associated regulation of the majority of *Chlamydomonas* MAPKs during the cell cycle, implying functional involvement in S/M-phase entry.

## Discussion

MAPK pathways are key signal transduction mechanisms in apparently all eukaryotes. Besides animals, fungi and plants they were also identified in protists, such as *Dictyostelium discoideum*^26^ or *Naegleria gruberi*^5^, implying the formation of MAPK signalling in the last eukaryotic common ancestor (LECA). However, their evolution took markedly different paths in opisthokonts and plants. Early divergence of the green algal and land plant lineages can be utilised to infer common ancestral genes of the plant kingdom. Therefore, in order to elucidate the early evolution of the MAPK gene family in plants, we identified and analysed MAPK sequences in 13 algal genomes, which represent the major lineages within Chlorophyta, the photosynthetic green microalgae (Table 1).

Separation of the land plant lineage is estimated to have occurred about 7–800 million years ago. The large and diverse set of extant algal species have emerged during this period, therefore comparative analysis across green algae and flowering plants, where MAPK phylogeny is well established, can provide insight into the early evolution of the gene family in the plant kingdom. In principle, MAPK types that are found across algae and land plants must have been formed prior to the separation of these lineages, and conversely, MAPK types that are missing in particular lineages only, must represent a lineage-specific gene loss that occurred since the separation of that lineage. Furthermore, however divergent, plant MAPKs are most closely related to a single type of animal MAPKs, ERK. Its main function is in positive regulation of S-phase entry, but it is unclear whether this function is original or if it was preserved in plants and protists.

Algal species have relatively low numbers of MAPKs (Table 2), and phylogenetic analysis revealed that canonical MAPKs form three major clades throughout green algae (A/B, C, D). Phylogenetic relationships of the sequences within each group represent well the phylogenetic relationships of the corresponding species (Figs 1 and 4), supporting their origin from a common ancestor. In most species there is one MAPK of each type, exceptions are gene losses and a lineage-specific duplication of the D-type kinase in Chlorophyceae (*Chlamydomonas*, *Gonium*, *Volvox*). These findings mean that in an early common ancestor of the plant kingdom three different MAPK genes were the founding members of the plant MAPK gene family, and all plant MAPKs emerged from consecutive duplications of these three basic types, eventually leading to the presence of about twenty MAPKs in flowering plants, forming four phylogenetic groups. Group A and B MAPKs thus emerged through the separation of the ancient A/B group.

Interestingly, a number of species with losses of A/B or C group members are viable, suggesting a degree of functional redundancy of the two early TEY-type MAPKs. In contrast, in Arabidopsis loss of single (MPK4) or close paralogous (MPK3/6) MAPK genes leads to severe developmental defects or lethality, indicating marked functional separations. Our knowledge on TDY MAPKs is rather limited, even though they form the largest phylogenetic MAPK group (group D)^21^. Interestingly, a duplication of the ancestral TDY kinase also occurred in some Chlorophyceae species. These species have both the largest genome sizes and gene numbers among the studied algae, in line with the notion that MAPK gene family expansion can be associated with increasing genome complexity.

Neofunctionalisation of the basic MAPK types is also supported by an evident increase of Ka/Ks ratios with increasing phylogenetic distances. Nevertheless, the overall Ka/Ks ratios are very low, because most MAPK sequences consist almost entirely of a kinase domain, which, as a catalytic domain, must be under strong purifying selection. Therefore, besides regions outside of the kinase domain, positive selection forces may as well act on small, functionally less stringent sites within the kinase domain, to which we have demonstrated conspicuous examples in the recently duplicated paralogues of group D MAPKs in Chlorophyceae (Fig. 5C,D). These events imply that recent duplication provides a window of opportunity for restricted positive selection to drive accelerated functional divergence.

The species included in this work live in a wide range of ecological conditions, and our results also reveal that the number of MAPKs in algae is not defined by life style or habitat, despite the important role of MAPKs in environmental signalling in flowering plants. For example, the endoparasitic *Helicosporidium*, which lives in a highly stable environment contains one of each MAPK type, like most of the free-living species, while the A/B-type MAPK is lost in *Coccomyxa subellipsoidea*, which lives under the extreme conditions of Antarctica.

The atypical MAPK, ERK8 is associated with cell cycle regulation in mammals. Interestingly, this kinase type was also identified in protists^27^, implying its formation prior to the separation of the major eukaryotic lineages, but it is missing from flowering plants. Accordingly, ERK8 homologs are present throughout algae, although it appears to be also dispensable, as it is lost in Prasinophyceae species with the most compacted genomes. In addition, the *Chlorella variabilis* ERK8-like sequence contains a 14-amino-acid insertion within the catalytic loop, raising the possibility that it is not a functional kinase. Subsequent loss of ERK8 in plants may indicate the evolution of plant-specific cell cycle regulatory mechanisms.

Signalling outputs of MAPK pathways are defined by the protein substrates they phosphorylate^28^. MAPK-interacting proteins, such as upstream MKKs, regulatory phosphatases and downstream substrates bind through short dedicated docking motifs^20^. The most common binding mechanism is between the D-site of various interactors and the corresponding CD (common docking) site of MAPKs (Fig. 3). The CD motif is well conserved across kingdoms and algal A/B group MAPKs possess highly conserved CD motifs, whereas the canonical pattern is systematically modified in the other groups^5^. Such CD site modifications may indicate a drift in substrate binding specificities. Accordingly, certain group-specific substitutions of flowering plants are not present in algae, such as E to N in position 7 in group C or D/E to K in position 3 in group D. These remarkable remnants of the canonical motif throughout algae fit well with the notion of specificity drift, indicating an earlier stage of the process in algae.

Unfortunately, our current knowledge on substrate proteins of C or D group MAPKs is very limited^29,30^. In light of the drift, identification of the corresponding D-site variants would be very informative in terms of the coevolution of short binding motifs. Such knowledge has important implications for *in silico* prediction of interactors through motif search, e.g.^31^.

The remarkable D to R substitution (position 6) of group D MAPKs results in a negative to positive charge swap. Such drastically altered binding surface must mean a substantially altered binding mechanism. This trend is a striking contrast to the evolution of animal MAPKs, where the CD motifs of the various MAPK types have remained highly conserved, despite the higher degree of divergence of the different MAPK pathways and the emergence of different phosphosite motifs. In plants, on the other hand, while novel MAPK types have not evolved, the CD motifs of the plant-type ERK variants have undergone pronounced alterations. CD site alterations also raise questions about the means of docking by interactors other than substrates, especially MKKs, which will require further studies.

ERK, the most ancient MAPK type occurring throughout eukaryotes is the closest relative of plant MAPKs. Although its function in animal cell cycle regulation has been established, its association with the cell cycle is much less explicit in plants and protists. ERK homologues regulate developmental processes both in plant and protist models^7,26^, but, of the various aspects of the cell cycle they have so far been only associated with one specific process, the cytokinesis in plants^32^. Given its evolutionary position and wealth of biochemical and physiological features, the model algal species, *Chlamydomonas reinhardtii* provides a unicellular plant experimental system, which is especially suitable for cell cycle studies^33^. Gene expression data in synchronised cultures revealed similar transient induction of *MAPK* gene expression during alternating S/M phases as that of the cell cycle regulatory kinase, *CDKB* (Fig. 6). The function of CDKB, an essential gene in *C. reinhardtii*, lies in regulation of mitosis and promoting S phase^25,34^. The correlation between the two expression patterns suggest a possible function in the regulation S and/or M phase that may be similar to that of mammalian ERK1/2.

Despite the overall similarities, there are also some differences between the MAPK expression profiles in the previous transcriptome analysis^22^ and our qPCR assays. Besides the methodology, the observed differences may be the consequence of the differences in culturing conditions, which may indicate environmental modulation of expression, and suggest a potential dual role of MAPKs both in cell cycle regulation and in environmental response.

We are not aware of any functional studies linking MAPKs to cell cycle regulation in algae. It has been demonstrated that pharmacological inhibition of MAPK signalling is feasible approach in microalgae^35^ and various mutant collections are also available, e.g.^18^. Therefore, cell cycle regulatory function of algal MAPKs is amenable for directed functional studies. Nevertheless, the concept is in good agreement with the notion that the common ancestor of eukaryotic MAPKs was a cell cycle regulator kinase.

In light of the separation of stress-signalling MAPK pathways in opisthokonts and the parallel expansion of the ERK-like genes in higher plants, it seems reasonable to propose that an original cell cycle regulatory MAPK pathway was co-opted for stress signalling in the plant lineage. In contrast to animals, plant development is mainly postembryonic and highly flexible to environmental conditions, due to sessile life style. Thus, separating cell cycle and developmental regulation from stress responses is more reasonable in animals, while higher plants could utilise the ancestral ERK modules to evolve a highly complex network through extensive duplications. The plant-specific ERK-like network might have also made ERK8 redundant, leading to an eventual loss.

In conclusion, our work provides a comprehensive framework for the early evolution of the MAPK gene family in the plant kingdom through comparative genomic analysis of algal MAPK sequences. Our results reveal the presence of three phylogenetic MAPK types across Chlorophyta, strongly implying the evolution of plant MAPKs from three ancestral MAPK forms. Atypical MAPKs related to mammalian ERK8 are ubiquitously present, despite their absence in flowering plants, demonstrating the emergence of an ancestral ERK8 kinase in a common eukaryotic ancestor, and its functional relevance in the algal lineages. Our work also highlights the scarcity of functional knowledge regarding cellular signalling, and MAPK pathways in particular, in microalgae. Functional knowledge of algal MAPK signalling would not only advance better understanding of the evolution of this important signalling mechanism in the plant kingdom, but can also have practical implications in the emerging algal bioindustry.

## Materials and Methods

### Data collection of thirteen algal genomes

For sequence analysis thirteen algal reference genome assemblies were retrieved from NCBI Assembly (https://www.ncbi.nlm.nih.gov/assembly), both at the genome and proteome levels. These are *Bathycoccus prasinos* (GCA_002220235.1); *Ostreococcus lucimarinus* CCE9901 (GCF_000092065.1); *Ostreococcus tauri* (GCF_000214015.2); *Micromonas commoda* (GCF_000090985.2); *Micromonas pusilla* CCMP1545 (GCF_000151265.2); *Auxenochlorella protothecoides* (GCF_000733215.1); *Chlorella variabilis* (GCF_000147415.1); *Helicosporidium sp*. ATCC 50920 (GCA_000690575.1); *Coccomyxa subellipsoidea* C-169 (GCF_000258705.1); *Chlamydomonas reinhardtii* (GCF_000002595.1); *Monoraphidium neglectum* (GCF_000611645.1); *Gonium pectoral*e (GCA_001584585.1); *Volvox carteri* f. nagariensis (GCF_000143455.1).

### Identification of MAPKs

To identify MAP kinase sequences within the proteomes of the 13 algal species we built a Hidden Markov Model (HMM) profile based on multiple alignments of the kinase domain sequences of the 20 *Arabidopsis thaliana* MAPK sequences^21^, using *hmmbuild*. The resulting protein collections were subsequently scanned with *hmmscan* packages of HMMER 3.0^36^. Canonical MAPKs were identified within the generated list of sequences by individual search for the presence of the characteristic TxY and flanking motifs.

### Sequence alignments and phylogenetic reconstruction

Actin sequences were used to confirm the taxonomic hierarchy of 13 algal species and selected MAP kinase sequences for the analysis of species-related relationships between MAP kinase groups. The actins at amino acid level (AA_actins_), and the 48 identified MAP kinase sequences (full-length amino acid (AA_mapk-fl_) and nucleotide (NT_mapk-fl_), and kinase-domain-only (AA_mapk-kd_) amino acid sequences) from the thirteen algal species were aligned using a MUSCLE alignment method and inferred using Maximum-likelihood phylogenetic tree by MEGA6 software package^37^. Based on the Bayesian Information Criterion (BIC) the best-fit substitution patterns were chosen for each phylogenetic reconstruction. The most fitting substitution pattern for classification of the investigated algal species was described by the Le_Gascuel_2008 model (LG + G). For the phylogenetic reconstruction from the AA_mapk-fl_ and AA_mapk-kd_ MAP kinase sequences were also described by the LG + G model, while the Tamura 3-parameter model (T92 + G + I) was used in case of reconstruction from the MAP kinase NT_mapk-fl_ sequences. Cyclin-dependent kinase (CDKG1), a related, non-MAPK protein kinase sequence of *Chlamydomonas reinhardtii* was used as outgroup. One thousand bootstrap pseudo-replicates were used to test the reliability of the inferred trees.

### Ka/Ks calculation

1175 pairwise alignments were generated between the 48 algal MAP kinase sequences using the MUSCLE method of the MEGA6 software package^37^, then AXT format files were prepared using a self-made script. Ka (nonsynonymous) and Ks (synonymous) ratios were calculated with the KaKs_Calculator2.0 software^38^, using the Model Average (MA) method.

### Sliding window analysis

Sliding window approach for estimating sequence divergence was carried out to analyse the Ka (nonsynonymous) and Ks (synonymous) rates between representative proteins from orthologous *C. reinhardtii* and *V. carteri* group A/B, C and D MAP kinases. The *SPLIT* and *PLOT* modules of KaKs_Calculator2.0^38^ were used for formatting datasets (window size = 51 bp; step size = 9 bp) and to visualize the results.

### Experimental organism, culture growth conditions, cell cycle synchronization and analysis

*Chlamydomonas reinhardtii* (wild type 21gr, cc-1690) cells were synchronized for at least three cycles of alternating 13 h light and 11 h dark periods (13:11 h) at 30 °C in custom-made 300 ml tubes in HS medium aerated with 2% (v/v) CO_2_. The tubes were illuminated by fluorescent tubes OSRAM L36/41; the light intensity at the surface of culture vessels was 490 µmol·m^−2^·s^−1^. The cell concentration during the entire synchronization procedure was kept under 2 × 10^6^ cells.ml^−1^ by dilution. For the experiment, the culture was diluted to a starting concentration of 1 × 10^6^ cells/ml and cultivated in 2.5 l plan-parallel vessels.

Attainment of commitment point was evaluated in hourly taken aliquots. Each 1 ml aliquot was spread on HSM plate and cultivated at 30 °C in the dark until the cell division in the slowest dividing culture was completed. The percentage of cells undivided or divided into 2, 4 or 8 daughter cells were calculated and plotted as a function of time. The percentage of cells that completed protoplast fission into two, four and eight cells and cell division was counted in samples fixed by Lugol solution (1 g I, 5 g KI, 100 ml H_2_O) at a final concentration 10 μl of Lugol solution per 1 ml of cell suspension and plotted as a function of time. Observations in transmitted light microscopy were carried out using an Olympus BX51 microscope (www.olympus-global.com) equipped with a CCD camera (F-ViewII).

### Quantitative RT-PCR

Cell pellets containing 2 × 10^7^ cells were harvested during the cell cycle, mixed with 1 ml of DNA/RNA Shield (Zymo Research, www.zymoreasearch.com) and stored at −20 °C. RNA was isolated by ZR Plant RNA MiniPrep Kit (Zymo Research, www.zymoreasearch.com) according to the manufacturer’s instructions. cDNA synthesis was carried using random hexamers in 1 µg of RNA by RevertAid First Strand cDNA Synthesis Kit (Thermo Fisher Scientific^TM^, www.thermofisher.com). For each quantitative RT-PCR reaction, 1 μl of cDNA was combined with primers and appropriately diluted in Maxima™ SYBR Green qPCR Master Mix (Thermo Fisher Scientific^TM^, www.thermofisher.com). For amplification of *CDKB1* transcripts the following primers were used: forward primer 5′-GCTGCCTTCTATGCACATCA-3′, reverse primer: 5′-TCTCGTGCGTGTAGCTCTTG-3′, other primers were following: *MPK2* forward primer 5′-CGGTCTGAGAGCATGCGC-3′, reverse primer: 5′-CGCCAGCACCTACTAACACCC-3′, *MPK3* forward primer 5′-GCTGGCCATCGACCTAAT-3′, reverse primer 5′-ATCACGAAATGTCTGGCG-3′*, MPK4* forward primer 5′-TCATGTCTGGCGGCTTGTC-3′, reverse primer 5′-GCCATGCACGTTCCTTGT-3′, *MPK5* forward primer 5′-CGACCACCAACACGCACTAC-3′, reverse primer 5′-CATGAAGCCAAACACGCTCG-3′, *MPK6* forward primer 5′-TCAACCACTACGGCCACAAG-3′, reverse primer 5′- TGCGTGAACACAAGACCG-3′*, MPK8* forward primer 5′-TCACCAAGACGGCGCTG-3′, reverse primer 5′-CCGCCCGTCCATCGTC-3′*, GBLP* forward primer 5′-GTCATCCACTGCCTGTGCTTCT-3′, reverse primer 5′-GGCCTTCTTGCTGGTGATGTT-3′. Quantitative RT-PCR was performed in Rotor-Gene RG-3000 (Corbett Science, www.corbettlifescience.com) under the following conditions: initial denaturation 10 min at 95 °C followed by 45 cycles of amplification (20 sec at 95 °C, 20 sec at 55 °C, 30 sec at 72 °C). PCR was performed in technical duplicates, differing by less than 5% between each other; the experiments were repeated in at least three biological replicates. To ensure that no primer-dimers were present, a melting curve was followed for each PCR. The results were normalized by NORMA-Gene macro^23^. We used this normalization because, in our hands, all other widely used reference genes such as G-protein β-subunit-like protein (GBLP), actin, tubulin or ubiquitin^39,40^, showed a cell cycle oscillating expression.

## Supplementary information

Supplementary Materials
