# Supplementary material for: Early Evolution of the Mitogen-Activated Protein Kinase Family in the Plant Kingdom
Source: Sci Rep. 2019 Mar 11;9:4094. doi: 10.1038/s41598-019-40751-y (PMC6411719; doi:10.1038/s41598-019-40751-y)
Supplement: Supplementary file 1 — Supplementary Materials [file 41598_2019_40751_MOESM1_ESM.pdf]

Early Evolution of the Mitogen-Activated Protein Kinase Family in the Plant Kingdom

Balázs Kalapos[1,2], Monika Hlavová[3], Tímea V. Nádai[1,2], Gábor Galiba[1,2], Kateřina Bišová[3] and Róbert Dóczi[1]

[1] Institute of Agriculture, Centre for Agricultural Research of the Hungarian Academy of Sciences, H-2462 Martonvásár, Brunszvik u. 2, Hungary

[2] Festetics Doctoral School, Georgikon Faculty, University of Pannonia, 8360 Keszthely, Hungary

[3] Centre Algatech, Institute of Microbiology Academy of Sciences of the Czech Republic, Opatovicky mlyn, CZ 379 81, Třeboň, Czech Republic

* Correspondence to: Róbert Dóczi, doczi.robert@agrar.mta.hu

Authors' e-mail addresses:

Balázs Kalapos: kalapos.balazs@agrar.mta.hu
Monika Hlavová: hlavova@alga.cz
Tímea V. Nádai: nadai.timea@agrar.mta.hu
Gábor Galiba: galiba.gabor@agrar.mta.hu
Kateřina Bišová: bisova@alga.cz
Róbert Dóczi: doczi.robert@agrar.mta.hu

Supplementary Materials

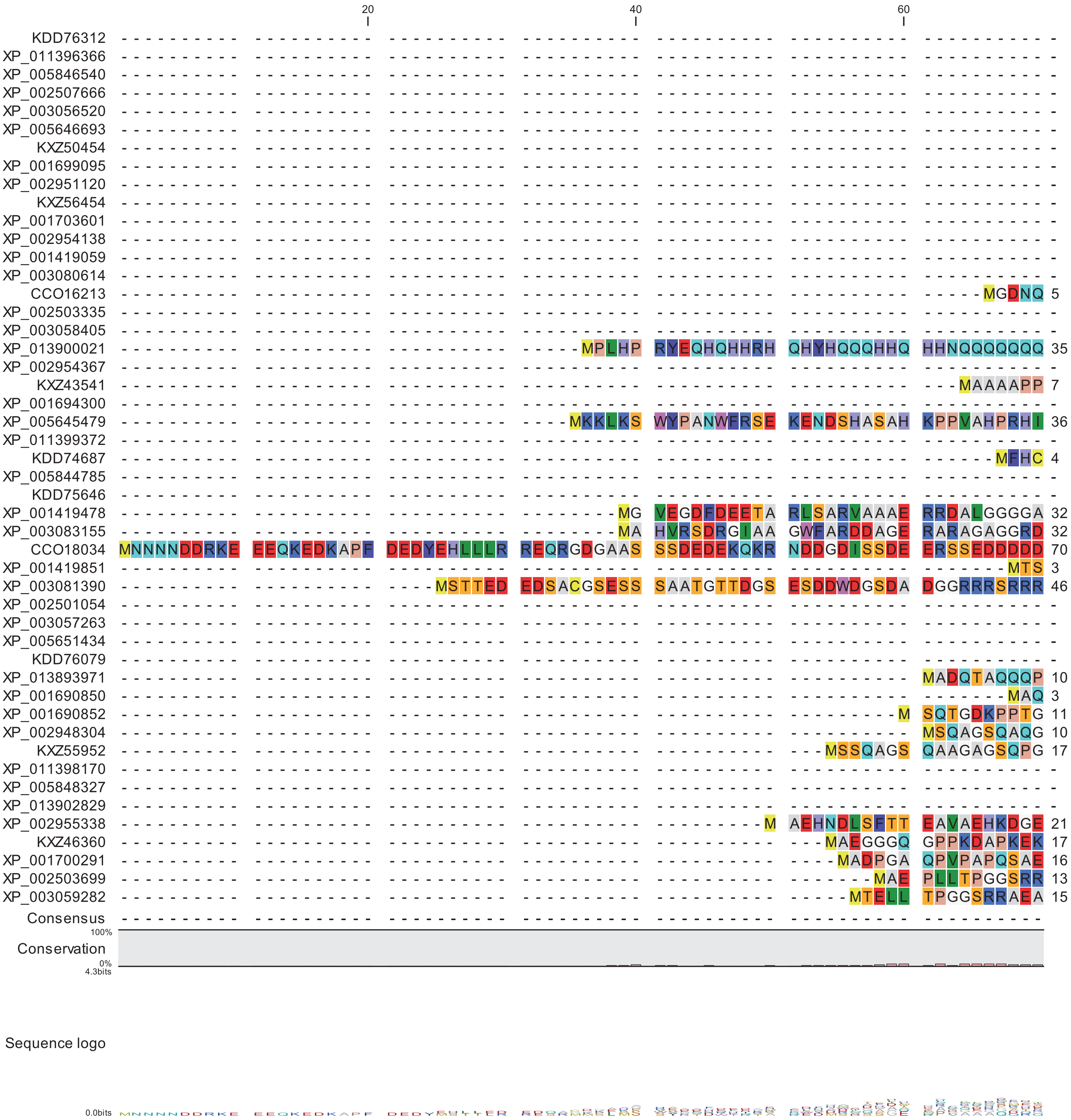

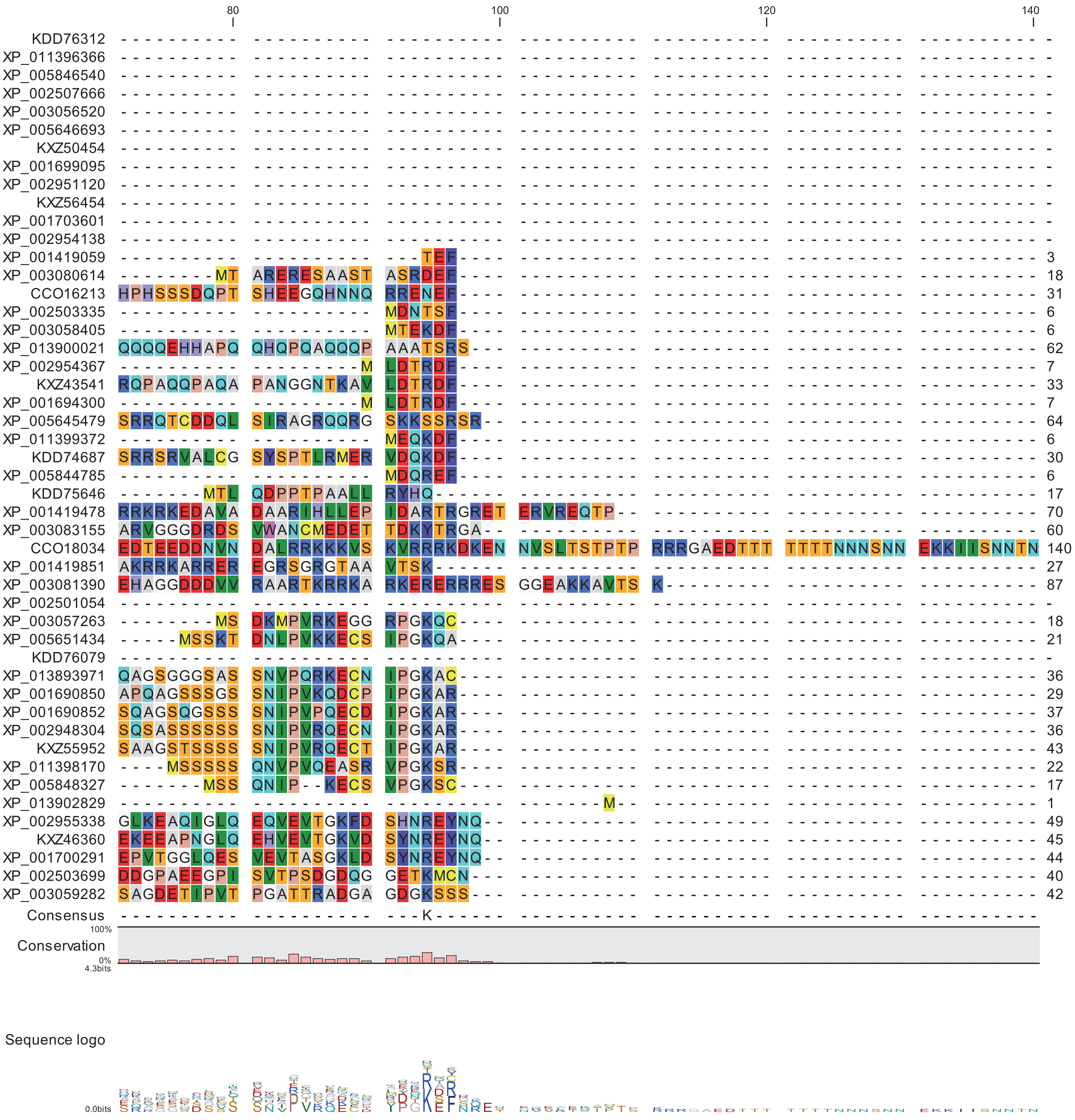
80
100
120
140
KDD76312
XP_011396366
XP_005846540
XP_002507666
XP_003056520
XP_005646693
KXZ50454
XP_001699095
XP_002951120
KXZ56454
XP_001703601
XP_002954138
XP_001419059
XP_003080614
CCO16213
XP_002503335
XP_003058405
XP_013900021
XP_002954367
KXZ43541
XP_001694300
XP_005645479
XP_011399372
KDD74687
XP_005844785
KDD75646
XP_001419478
XP_003083155
CCO18034
XP_001419851
XP_003081390
XP_002501054
XP_003057263
XP_005651434
KDD76079
XP_013893971
XP_001690850
XP_001690852
XP_002948304
KXZ55952
XP_011398170
XP_005848327
XP_013902829
XP_002955338
KXZ46360
XP_001700291
XP_002503699
XP_003059282
Consensus
100%
Conservation
0%
4.3bits
Sequence logo
0.0bits

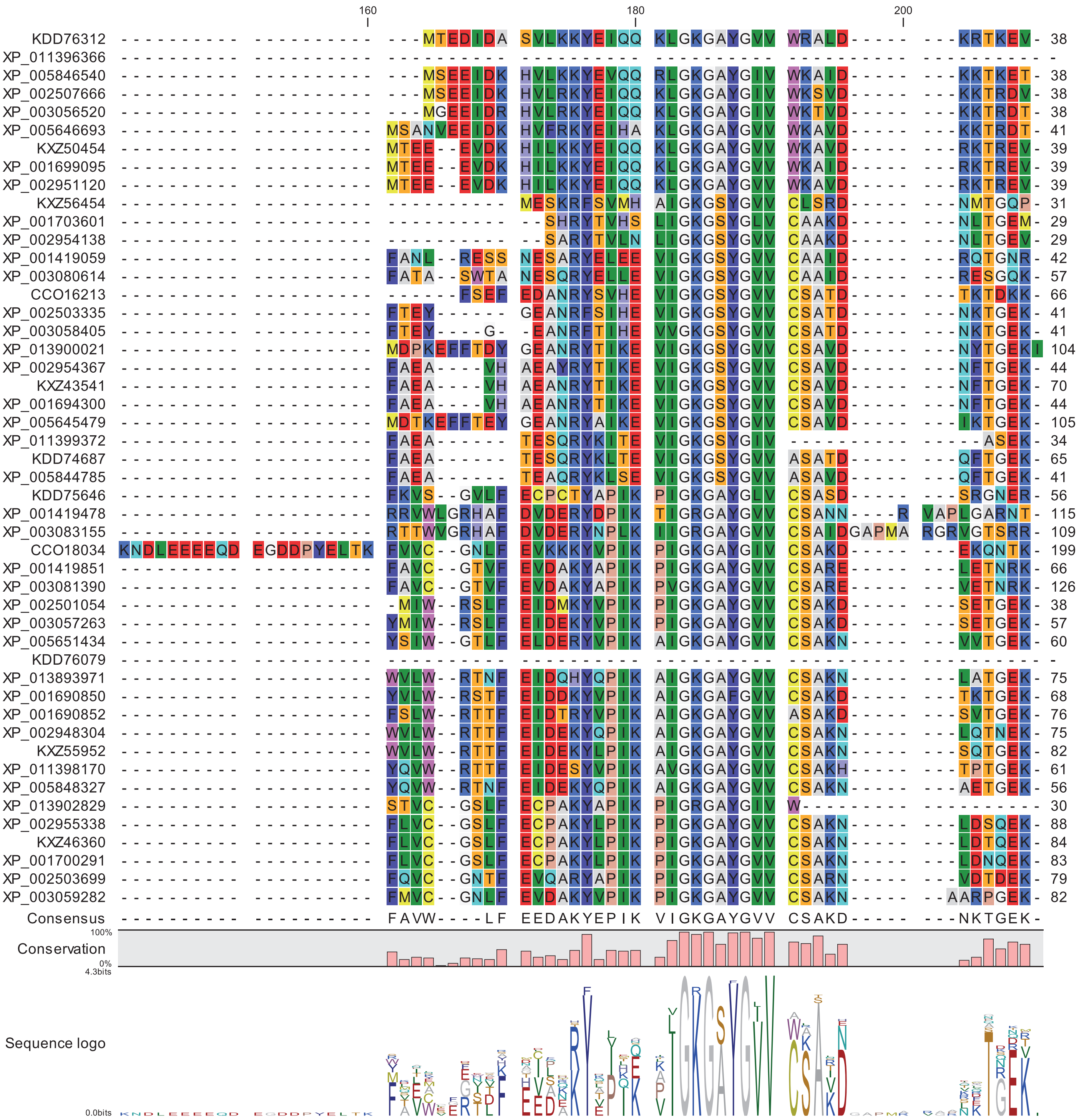
160
180
200
KDD76312
XP_011396366
XP_005846540
XP_002507666
XP_003056520
XP_005646693
KXZ50454
XP_001699095
XP_002951120
KXZ56454
XP_001703601
XP_002954138
XP_001419059
XP_003080614
CCO16213
XP_002503335
XP_003058405
XP_013900021
XP_002954367
KXZ43541
XP_001694300
XP_005645479
XP_011399372
KDD74687
XP_005844785
KDD75646
XP_001419478
XP_003083155
CCO18034
XP_001419851
XP_003081390
XP_002501054
XP_003057263
XP_005651434
KDD76079
XP_013893971
XP_001690850
XP_001690852
XP_002948304
KXZ55952
XP_011398170
XP_005848327
XP_013902829
XP_002955338
KXZ46360
XP_001700291
XP_002503699
XP_003059282
Consensus
Conservation
100%
0%
4.3bits
Sequence logo
0.0bits

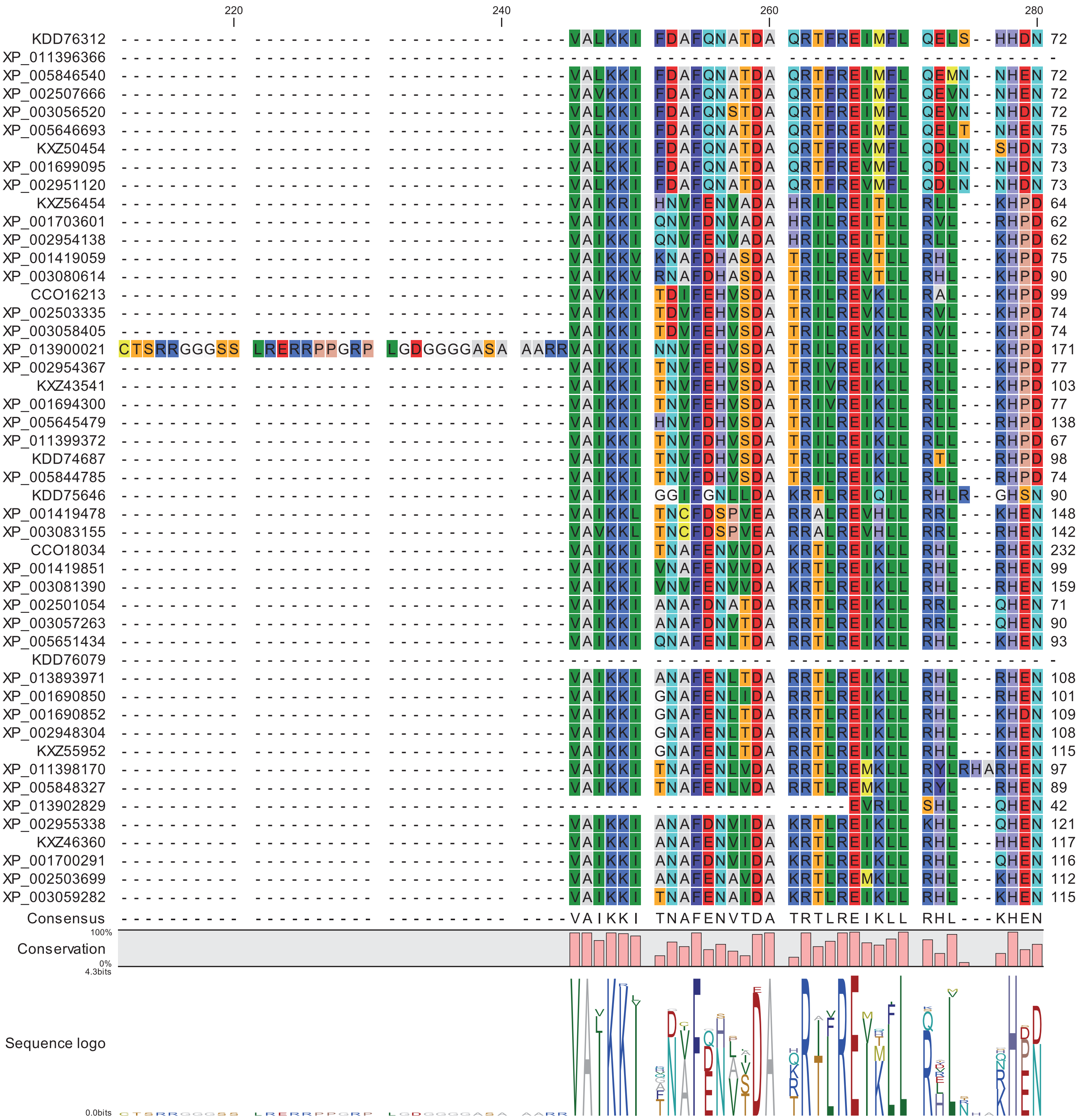

220
240
260
280
KDD76312 ---------- ---------- ---------- ----VALKKI FDAFQNATDA QRTFREIMFL QELS--HHDN 72
XP_011396366 ---------- ---------- ---------- ---------- ---------- ---------- ---------- -
XP_005846540 ---------- ---------- ---------- ----VALKKI FDAFQNATDA QRTFREIMFL QEMN--NHEN 72
XP_002507666 ---------- ---------- ---------- ----VAVKKI FDAFQNATDA QRTFREIMFL QEVN--NHEN 72
XP_003056520 ---------- ---------- ---------- ----VALKKI FDAFQNSTDA QRTFREIMFL QEVN--NHDN 72
XP_005646693 ---------- ---------- ---------- ----VALKKI FDAFQNATDA QRTFREIMFL QELT--NHEN 75
KXZ50454 ---------- ---------- ---------- ----VALKKI FDAFQNATDA QRTFREVMFL QDLN--SHDN 73
XP_001699095 ---------- ---------- ---------- ----VALKKI FDAFQNATDA QRTFREVMFL QDLN--NHDN 73
XP_002951120 ---------- ---------- ---------- ----VALKKI FDAFQNATDA QRTFREVMFL QDLN--NHDN 73
KXZ56454 ---------- ---------- ---------- ----VAIKRI HNVFENVADA HRILREITLL RLL---KHPD 64
XP_001703601 ---------- ---------- ---------- ----VAIKKI QNVFDNVADA HRILREITLL RVL---RHPD 62
XP_002954138 ---------- ---------- ---------- ----VAIKKI QNVFENVADA HRILREITLL RLL---KHPD 62
XP_001419059 ---------- ---------- ---------- ----VAIKKV KNAFDHASDA TRILREVTLL RHL---KHPD 75
XP_003080614 ---------- ---------- ---------- ----VAIKKV RNAFDHASDA TRILREVTLL RHL---KHPD 90
CCO16213 ---------- ---------- ---------- ----VAVKKI TDIFEHVSDA TRILREVKLL RAL---KHPD 99
XP_002503335 ---------- ---------- ---------- ----VAIKKI TDVFEHVSDA TRILREVKLL RVL---KHPD 74
XP_003058405 ---------- ---------- ---------- ----VAIKKI TDVFEHVSDA TRILREVKLL RVL---KHPD 74
XP_013900021 CTSRRGGGSS LRERRPPGRP LGDGGGGASA AARRVAIKKI NNVFEHVSDA TRILREIKLL RLL---KHPD 171
XP_002954367 ---------- ---------- ---------- ----VAIKKI TNVFEHVSDA TRIVREIKLL RLL---KHPD 77
KXZ43541 ---------- ---------- ---------- ----VAIKKI TNVFEHVSDA TRIVREIKLL RLL---KHPD 103
XP_001694300 ---------- ---------- ---------- ----VAIKKI TNVFEHVSDA TRIVREIKLL RLL---KHPD 77
XP_005645479 ---------- ---------- ---------- ----VAIKKI HNVFDHVSDA TRILREIKLL RLL---RHPD 138
XP_011399372 ---------- ---------- ---------- ----VAIKKI TNVFDHVSDA TRILREIKLL RLL---RHPD 67
KDD74687 ---------- ---------- ---------- ----VAIKKI TNVFDHVSDA TRILREIKLL RTL---RHPD 98
XP_005844785 ---------- ---------- ---------- ----VAIKKI TNVFDHVSDA TRILREIKLL RLL---RHPD 74
KDD75646 ---------- ---------- ---------- ----VAIKKI GGIFGNLLDA KRTLREIQIL RHLR--GHSN 90
XP_001419478 ---------- ---------- ---------- ----VAIKKL TNCFDSPVEA RRALREVHLL RRL---KHEN 148
XP_003083155 ---------- ---------- ---------- ----VAVKKL TNCFDSPVEA RRALREVHLL RRL---RHEN 142
CCO18034 ---------- ---------- ---------- ----VAIKKI TNAFENVVDA KRTLREIKLL RHL---RHEN 232
XP_001419851 ---------- ---------- ---------- ----VAIKKI VNAFENVVDA KRTLREIKLL RHL---RHEN 99
XP_003081390 ---------- ---------- ---------- ----VAIKKI VNVFENVVDA KRTLREIKLL RHL---RHEN 159
XP_002501054 ---------- ---------- ---------- ----VAIKKI ANAFDNATDA RRTLREIKLL RRL---QHEN 71
XP_003057263 ---------- ---------- ---------- ----VAIKKI ANAFDNVTDA RRTLREIKLL RRL---QHEN 90
XP_005651434 ---------- ---------- ---------- ----VAIKKI QNAFENLTDA RRTLREIKLL RHL---KHEN 93
KDD76079 ---------- ---------- ---------- ---------- ---------- ---------- ---------- -
XP_013893971 ---------- ---------- ---------- ----VAIKKI ANAFENLTDA RRTLREIKLL RHL---RHEN 108
XP_001690850 ---------- ---------- ---------- ----VAIKKI GNAFENLIDA RRTLREIKLL RHL---RHEN 101
XP_001690852 ---------- ---------- ---------- ----VAIKKI GNAFENLTDA RRTLREIKLL RHL---KHDN 109
XP_002948304 ---------- ---------- ---------- ----VAIKKI GNAFENLTDA RRTLREIKLL RHL---KHEN 108
KXZ55952 ---------- ---------- ---------- ----VAIKKI GNAFENLTDA RRTLREIKLL RHL---KHEN 115
XP_011398170 ---------- ---------- ---------- ----VAIKKI TNAFENLVDA RRTLREMKLL RYLRHARHEN 97
XP_005848327 ---------- ---------- ---------- ----VAIKKI TNAFENLVDA RRTLREMKLL RYL---RHEN 89
XP_013902829 ---------- ---------- ---------- ---------- ---------- -----EVRLL SHL---QHEN 42
XP_002955338 ---------- ---------- ---------- ----VAIKKI ANAFDNVIDA KRTLREIKLL KHL---QHEN 121
KXZ46360 ---------- ---------- ---------- ----VAIKKI ANAFENVIDA KRTLREIKLL RHL---HHEN 117
XP_001700291 ---------- ---------- ---------- ----VAIKKI ANAFDNVIDA KRTLREIKLL RHL---QHEN 116
XP_002503699 ---------- ---------- ---------- ----VAIKKI ANAFENAVDA KRTLREMKLL RHL---KHEN 112
XP_003059282 ---------- ---------- ---------- ----VAIKKI TNAFENAIDA KRTLREIKLL RHL---KHEN 115
Consensus ---------- ---------- ---------- ----VAIKKI TNAFENVTDA TRTLREIKLL RHL---KHEN
Conservation
100%
0%
4.3bits
Sequence logo
0.0bits

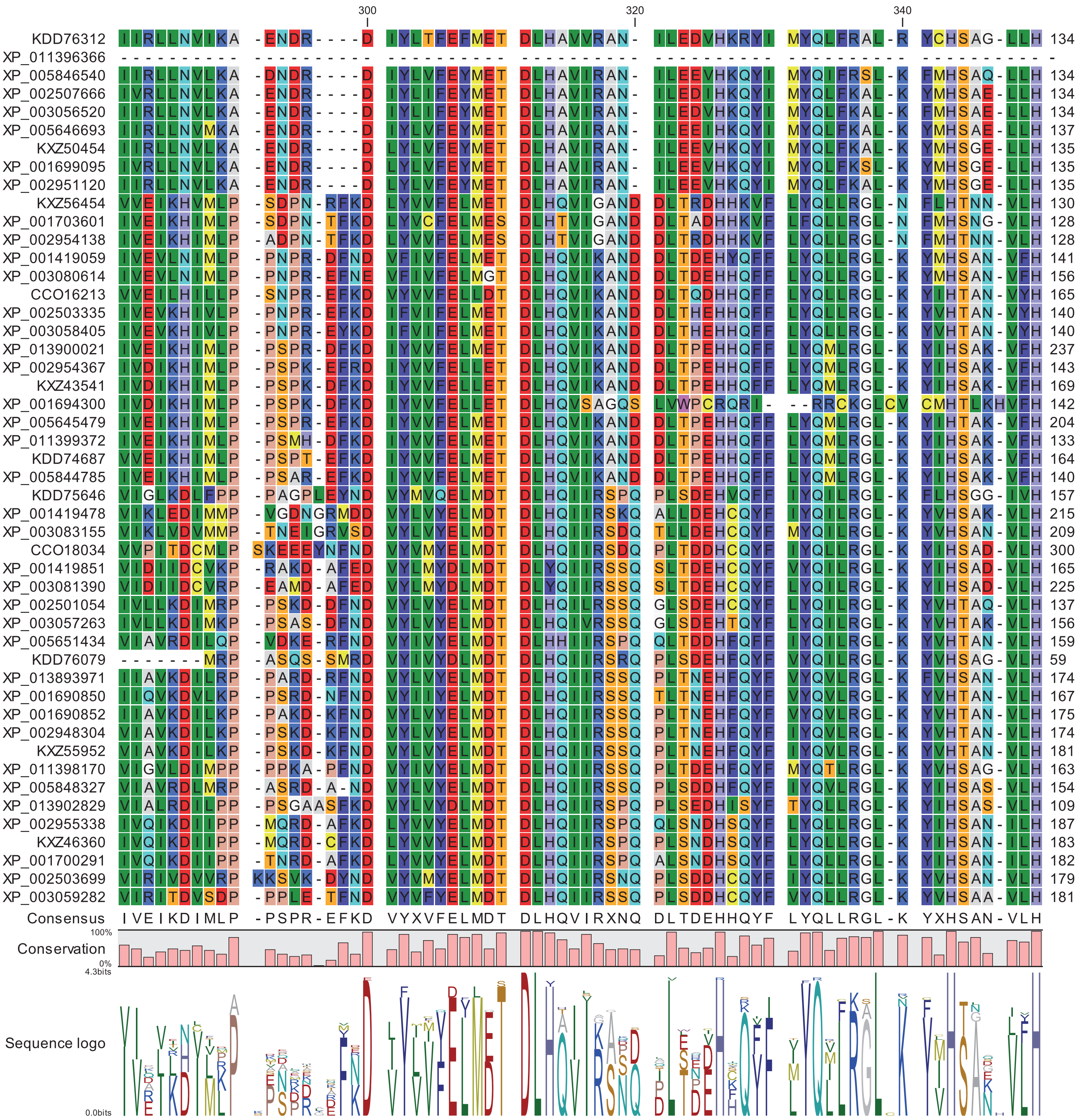
300
320
340
KDD76312
XP_011396366
XP_005846540
XP_002507666
XP_003056520
XP_005646693
KXZ50454
XP_001699095
XP_002951120
KXZ56454
XP_001703601
XP_002954138
XP_001419059
XP_003080614
CCO16213
XP_002503335
XP_003058405
XP_013900021
XP_002954367
KXZ43541
XP_001694300
XP_005645479
XP_011399372
KDD74687
XP_005844785
KDD75646
XP_001419478
XP_003083155
CCO18034
XP_001419851
XP_003081390
XP_002501054
XP_003057263
XP_005651434
KDD76079
XP_013893971
XP_001690850
XP_001690852
XP_002948304
KXZ55952
XP_011398170
XP_005848327
XP_013902829
XP_002955338
KXZ46360
XP_001700291
XP_002503699
XP_003059282
Consensus
IVEIKDIMLP -PSPR-EFKD VYXVFELMDT DLHQVIRXNQ DLTDEHHQYF LYQLLRGL-K YXHSAN-VLH
Conservation
100%
0%
4.3bits
Sequence logo
0.0bits

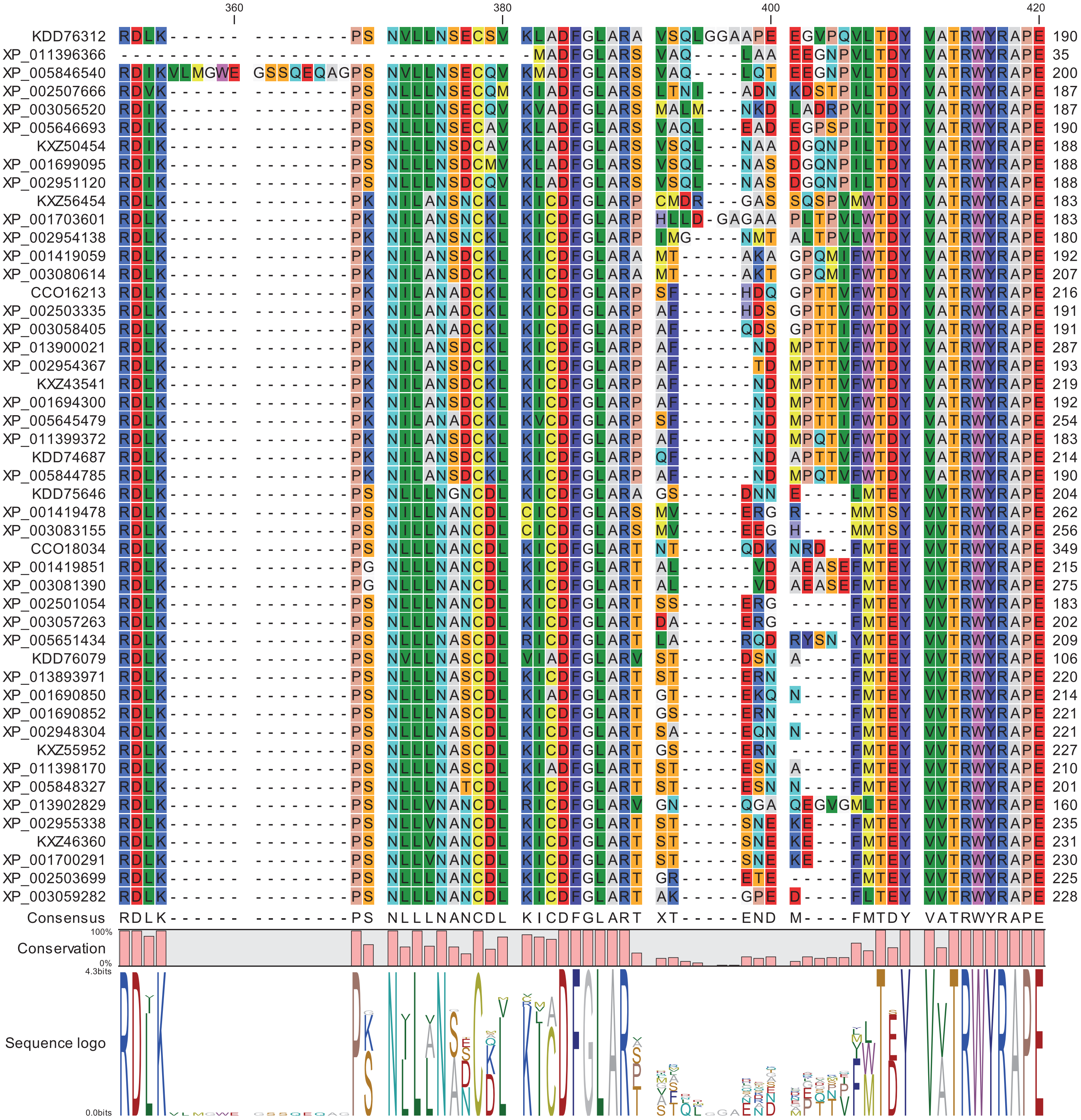
360 380 400 420
KDD76312 RDLK------ ---------PS NVLLNSECSV KLADFGLARA VSQLGGAAPE EGVPQVLTDY VATRWYRAPE 190
XP_011396366 ---------- ---------- ---------- -MADFGLARS VAQ----LAA EEGNPVLTDY VATRWYRAPE 35
XP_005846540 RDIKVLMGWE GSSQEQAGPS NVLLNSECQV KMADFGLARS VAQ----LQT EEGNPVLTDY VATRWYRAPE 200
XP_002507666 RDVK------ ---------PS NLLLNSECQM KIADFGLARS LTNI---ADN KDSTPILTDY VATRWYRAPE 187
XP_003056520 RDIK------ ---------PS NLLLNSECQV KVADFGLARS MALM---NKD LADRPVLTDY VATRWYRAPE 187
XP_005646693 RDIK------ ---------PS NLLLNSECAV KLADFGLARS VAQL---EAD EGPSPILTDY VATRWYRAPE 190
KXZ50454 RDIK------ ---------PS NLLLNSDCAV KLADFGLARS VSQL---NAA DGQNPILTDY VATRWYRAPE 188
XP_001699095 RDIK------ ---------PS NLLLNSDCMV KLADFGLARS VSQL---NAS DGQNPILTDY VATRWYRAPE 188
XP_002951120 RDIK------ ---------PS NLLLNSDCQV KLADFGLARS VSQL---NAS DGQNPILTDY VATRWYRAPE 188
KXZ56454 RDLK------ ---------PK NILANSNCKL KICDFGLARP CMDR---GAS SQSPVMWTDY VATRWYRAPE 183
XP_001703601 RDLK------ ---------PK NILANSNCKL KICDFGLARP HLLD-GAGAA PLTPVLWTDY VATRWYRAPE 183
XP_002954138 RDLK------ ---------PK NILANSNCKL KICDFGLARP IMG----NMT ALTPVLWTDY VATRWYRAPE 180
XP_001419059 RDLK------ ---------PK NILANSDCKL KICDFGLARA MT-----AKA GPQMIFWTDY VATRWYRAPE 192
XP_003080614 RDLK------ ---------PK NILANSDCKL KICDFGLARA MT-----AKT GPQMIFWTDY VATRWYRAPE 207
CCO16213 RDLK------ ---------PK NILANADCKL KICDFGLARP SF-----HDQ GPTTVFWTDY VATRWYRAPE 216
XP_002503335 RDLK------ ---------PK NILANADCKL KICDFGLARP AF-----HDS GPTTVFWTDY VATRWYRAPE 191
XP_003058405 RDLK------ ---------PK NILANADCKL KICDFGLARP AF-----QDS GPTTIFWTDY VATRWYRAPE 191
XP_013900021 RDLK------ ---------PK NILANSDCKL KICDFGLARP AF------ND MPTTVFWTDY VATRWYRAPE 287
XP_002954367 RDLK------ ---------PK NILANSDCKL KICDFGLARP AF------TD MPTTVFWTDY VATRWYRAPE 193
KXZ43541 RDLK------ ---------PK NILANSDCKL KICDFGLARP AF------ND MPTTVFWTDY VATRWYRAPE 219
XP_001694300 RDLK------ ---------PK NILANSDCKL KICDFGLARP AF------ND MPTTVFWTDY VATRWYRAPE 192
XP_005645479 RDLK------ ---------PK NILANADCKL KVCDFGLARP SF------ND MPTTIFWTDY VATRWYRAPE 254
XP_011399372 RDLK------ ---------PK NILANSDCKL KICDFGLARP AF------ND MPQTVFWTDY VATRWYRAPE 183
KDD74687 RDLK------ ---------PK NILANSDCKL KICDFGLARP QF------ND APTTVFWTDY VATRWYRAPE 214
XP_005844785 RDLK------ ---------PK NILANSDCKL KICDFGLARP AF------ND MPQTVFWTDY VATRWYRAPE 190
KDD75646 RDLK------ ---------PS NLLLNGNCDL KICDFGLARA GS-----DNN E----LMTEY VVTRWYRAPE 204
XP_001419478 RDLK------ ---------PS NILLNANCDL CICDFGLARS MV-----ERG R----MMTSY VVTRWYRAPE 262
XP_003083155 RDLK------ ---------PS NILLNANCDL CICDFGLARS MV-----EEG H----MMTSY VVTRWYRAPE 256
CCO18034 RDLK------ ---------PS NLLLNANCDL KICDFGLART NT-----QDK NRD--FMTEY VVTRWYRAPE 349
XP_001419851 RDLK------ ---------PG NLLLNANCDL KICDFGLART AL------VD AEASEFMTEY VVTRWYRAPE 215
XP_003081390 RDLK------ ---------PG NLLLNANCDL KICDFGLART AL------VD AEASEFMTEY VVTRWYRAPE 275
XP_002501054 RDLK------ ---------PS NLLLNANCDL KICDFGLART SS-----ERG -----FMTEY VVTRWYRAPE 183
XP_003057263 RDLK------ ---------PS NLLLNANCDL KICDFGLART DA-----ERG -----FMTEY VVTRWYRAPE 202
XP_005651434 RDLK------ ---------PS NLLLNASCDL RICDFGLART LA-----RQD RYSN-YMTEY VVTRWYRAPE 209
KDD76079 RDLK------ ---------PS NVLLNASCDL VIADFGLARV ST-----DSN A----FMTEY VVTRWYRAPE 106
XP_013893971 RDLK------ ---------PS NLLLNASCDL KICDFGLART ST-----ERN -----FMTEY VVTRWYRAPE 220
XP_001690850 RDLK------ ---------PS NLLLNASCDL KIADFGLART GT-----EKQ N----FMTEY VVTRWYRAPE 214
XP_001690852 RDLK------ ---------PS NLLLNASCDL KICDFGLART GS-----ERN -----FMTEY VVTRWYRAPE 221
XP_002948304 RDLK------ ---------PS NLLLNASCDL KICDFGLART SA-----EQN N----FMTEY VVTRWYRAPE 221
KXZ55952 RDLK------ ---------PS NLLLNASCDL KICDFGLART GS-----ERN -----FMTEY VVTRWYRAPE 227
XP_011398170 RDLK------ ---------PS NLLLNASCDL KIADFGLART ST-----ESN A----FMTEY VVTRWYRAPE 210
XP_005848327 RDLK------ ---------PS NLLLNATCDL KICDFGLART ST-----ESN N----FMTEY VVTRWYRAPE 201
XP_013902829 RDLK------ ---------PS NLLVNANCDL RICDFGLARV GN-----QGA QEGVGMLTEY VVTRWYRAPE 160
XP_002955338 RDLK------ ---------PS NLLVNANCDL KICDFGLART ST-----SNE KE---FMTEY VVTRWYRAPE 235
KXZ46360 RDLK------ ---------PS NLLVNANCDL KICDFGLART ST-----SNE KE---FMTEY VVTRWYRAPE 231
XP_001700291 RDLK------ ---------PS NLLVNANCDL KICDFGLART ST-----SNE KE---FMTEY VVTRWYRAPE 230
XP_002503699 RDLK------ ---------PS NLLLNANCDL KICDFGLART GR-----ETE -----FMTEY VVTRWYRAPE 225
XP_003059282 RDLK------ ---------PS NLLLNANCDL KICDFGLART AK-----GPE D----FLTEY VVTRWYRAPE 228
Consensus RDLK------ ---------PS NLLLNANCDL KICDFGLART XT-----END M----FMTDY VATRWYRAPE
Conservation
100%
0%
4.3bits
Sequence logo
0.0bits

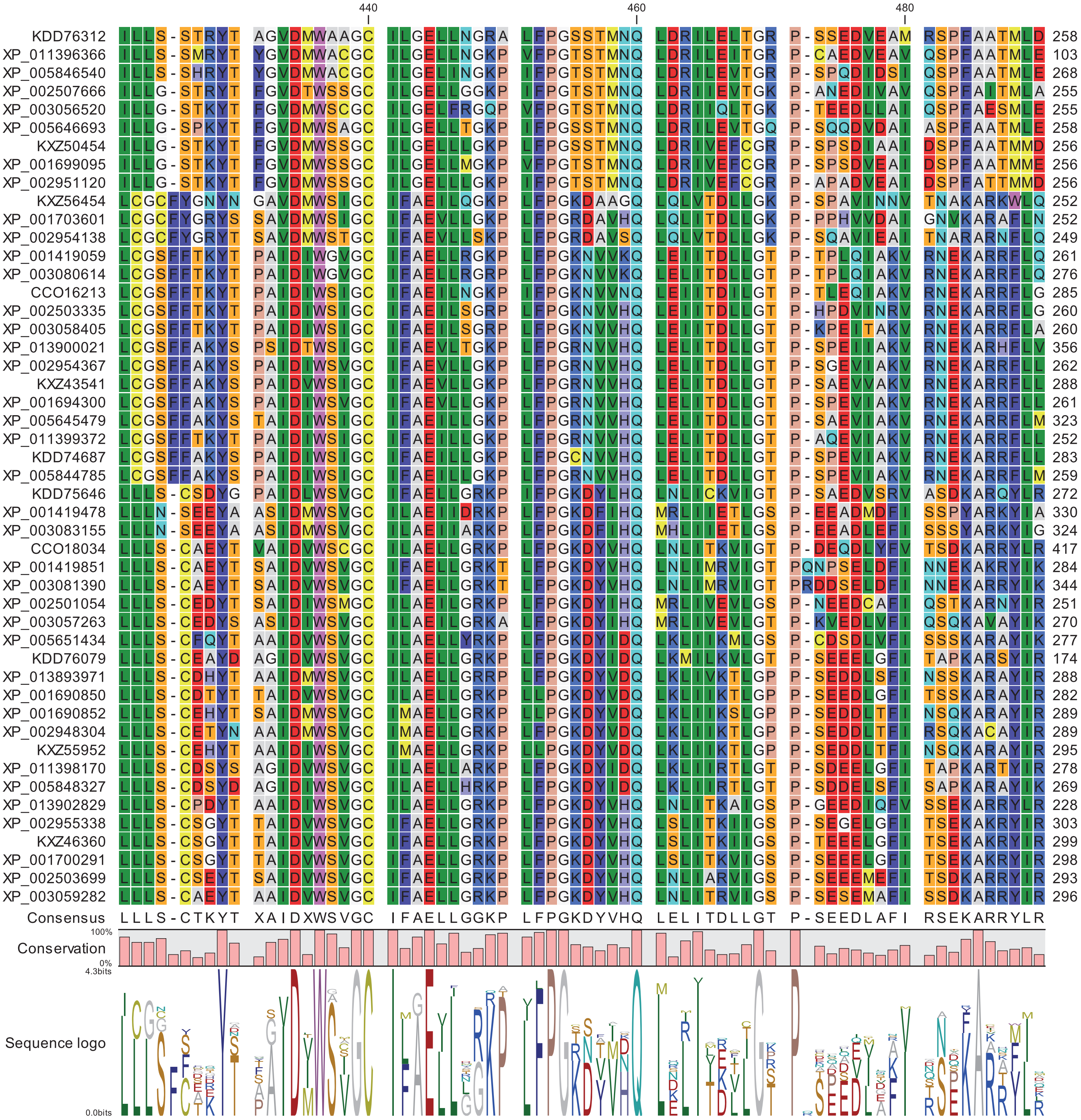
440 460 480
KDD76312 ILLS-STRYT AGVDMWAAGC ILGELLNGRA LFPGSSTMNQ LDRILELTGR P-SSEDVEAM RSPFAATMLD 258
XP_011396366 ILLS-SMRYT YGVDMWACGC ILGELLNGKP VFPGTSTMNQ LDRILEITGR P-CAEDVEAV QSPFAATMLE 103
XP_005846540 ILLS-SHRYT YGVDMWACGC ILGELINGKP IFPGTSTMNQ LDRILEVTGR P-SPQDIDSI QSPFAATMLE 268
XP_002507666 ILLG-STRYT FGVDTWSSGC ILGELLGGKP IFPGTSTMNQ LDRIIEVTGK P-ANEDIVAV QSPFAITMLA 255
XP_003056520 ILLG-STKYT FGVDMWSCGC ILGELFRGQP VFPGTSTMNQ LDRIIQLTGK P-TEEDLLAI QSPFAESMLE 255
XP_005646693 ILLG-SPKYT FGVDMWSAGC ILGELLTGKP IFPGSSTMNQ LDRILEVTGQ P-SQQDVDAI ASPFAATMLE 258
KXZ50454 ILLG-STKYT FGVDMWSSGC ILGELLLGKP LFPGSSTMNQ LDRIVEFCGR P-SPSDIAAI DSPFAATMMD 256
XP_001699095 ILLG-STKYT FGVDMWSSGC ILGELLMGKP VFPGTSTMNQ LDRIVEFCGR P-SPSDVEAI DSPFAATMME 256
XP_002951120 ILLG-STKYT FGVDMWSSGC ILGELLLGKP IFPGTSTMNQ LDRIVEFCGR P-APADVEAI DSPFATTMMD 256
KXZ56454 LCGCFYGNYN GAVDMWSIGC IFAEILQGKP LFPGKDAAGQ LQLVTDLLGK P-SPAVINNV TNAKARKWLQ 252
XP_001703601 LCGCFYGRYS SAVDMWSIGC IFAEVLLGKP LFPGRDAVHQ LQLVTDLLGK P-PPHVVDAI GNVKARAFLN 252
XP_002954138 LCGCFYGRYS SAVDMWSTGC IFAEVLLSKP LFPGRDAVSQ LQLVTDLLGK P-SQAVIEAI TNARARNFLQ 249
XP_001419059 LCGSFFTKYT PAIDIWGVGC IFAELLRGKP LFPGKNVVKQ LEIITDLLGT P-TPLQIAKV RNEKARRFLQ 261
XP_003080614 LCGSFFTKYT PAIDIWGVGC IFAELLRGRP LFPGKNVVKQ LELITDLLGT P-TPLQIAKV RNEKARRFLQ 276
CCO16213 LCGSFFTKYT PAIDIWSIGC IFAEILNGKP IFPGKNVVNQ LEIITDILGT P-TLEQIAKV RNEKARRFLG 285
XP_002503335 LCGSFFTKYT PAIDIWSIGC IFAEILSGRP LFPGKNVVHQ LEIITDLLGT P-HPDVINRV RNEKARRFLG 260
XP_003058405 LCGSFFTKYT PAIDIWSIGC IFAEILSGRP LFPGKNVVHQ LEIITDLLGT P-KPEITAKV RNEKARRFLA 260
XP_013900021 LCGSFFAKYS PSIDTWSIGC IFAEVLTGKP LFPGRNVVHQ LELITDLLGT P-SPEIIAKV RNEKARHFLV 356
XP_002954367 LCGSFFAKYS PAIDIWSVGC IFAEVLLGKP LFPGRNVVHQ LELITDLLGT P-SGEVIAKV RNEKARRFLL 262
KXZ43541 LCGSFFAKYS PAIDVWSIGC IFAEVLLGKP LFPGRNVVHQ LELITDLLGT P-SAEVVAKV RNEKARRFLL 288
XP_001694300 LCGSFFAKYS PAIDIWSVGC IFAEVLLGKP LFPGRNVVHQ LELITDLLGT P-SPEVIAKV RNEKARRFLL 261
XP_005645479 LCGSFFAKYS TAIDIWSIGC IFAEILLGKP LFPGRNVVHQ LELITDLLGT P-SAEVIAKV RNEKARRFLM 323
XP_011399372 LCGSFFTKYT PAIDIWSIGC IFAEILLGKP LFPGRNVVHQ LELITDLLGT P-AQEVIAKV RNEKARRFLL 252
KDD74687 LCGSFFAKYS PAIDIWSIGC IFAEILLGKP LFPGCNVVHQ LELITDLLGT P-SPEVIAKV RNEKARRFLL 283
XP_005844785 LCGSFFAKYS PAIDIWSIGC IFAEILLGKP LFPGRNVVHQ LELITDLLGT P-SPEVIAKV RNEKARRFLM 259
KDD75646 LLLS-CSDYG PAIDLWSVGC IFAELLGRKP IFPGKDYLHQ LNLICKVIGT P-SAEDVSRV ASDKARQYLR 272
XP_001419478 LLLN-SEEYA ASIDMWSVGC ILAEIIDRKP LFPGKDFIHQ MRLIIETLGS P-EEADMDFI SSPYARKYIA 330
XP_003083155 LLLN-SEEYA ASIDMWSVGC ILAEIIARKP LFPGKDFIHQ MHLIIETLGS P-EEADLEFI SSSYARKYIG 324
CCO18034 LLLS-CAEYT VAIDVWSCGC ILAELLGRKP LFPGKDYVHQ LNLITKVIGT P-DEQDLYFV TSDKARRYLR 417
XP_001419851 LLLS-CAEYT SAIDVWSVGC IFAELLGRKT LFPGKDYVHQ LNLIMRVIGT PQNPSELDFI NNEKAKRYIK 284
XP_003081390 LLLS-CAEYT SAIDVWSVGC IFAELLGRKT LFPGKDYVHQ LNLIMRVIGT PRDDSELDFI NNEKARRYIK 344
XP_002501054 LLLS-CEDYT SAIDIWSMGC ILAEILGRKP LFPGKDYIHQ MRLIVEVLGS P-NEEDCAFI QSTKARNYIR 251
XP_003057263 LLLS-CEDYS ASIDIWSVGC ILAEILGRKA LFPGKDYIHQ MRLIVEVLGT P-KVEDLVFI QSQKAVAYIK 270
XP_005651434 LLLS-CFQYT AAIDVWSVGC ILAELLYRKP LFPGKDYIDQ LKLIIKMLGS P-CDSDLVFI SSSKARAYIK 277
KDD76079 LLLS-CEAYD AGIDVWSVGC ILAELLGRKP LFPGKDYIDQ LKMILKVLGT P-SEEELGFI TAPKARSYIR 174
XP_013893971 LLLS-CDHYT AAIDMWSVGC ILAELLGRRP LFPGKDYVDQ LKLIVKTLGP P-SEDDLSFI NSSKARAYIR 288
XP_001690850 LLLS-CDTYT TAIDVWSVGC ILAELLGRKP LLPGKDYVDQ LKLIIKTLGP P-SEEDLGFI TSSKARAYIR 282
XP_001690852 LLLS-CEHYT SAIDMWSVGC IMAELLGRKP LLPGKDYVDQ LKLIIKSLGP P-SEDDLTFI NSQKARAYIR 289
XP_002948304 LLLS-CETYN AAIDMWSVGC IMAELLGRKP LFPGKDYVDQ LKLIIKTLGP P-SEDDLTFI RSQKACAYIR 289
KXZ55952 LLLS-CEHYT AAIDIWSVGC IMAELLGRKP LLPGKDYVDQ LKLIIKTLGP P-SEDDLTFI NSQKARAYIR 295
XP_011398170 LLLS-CDSYS AGIDVWSVGC ILAELLARKP LFPGKDYIDQ LKLIIRTLGT P-SDEELGFI TAPKARTYIR 278
XP_005848327 LLLS-CDSYD AGIDIWSVGC ILAELLHRKP LFPGKDYIDQ LKLIIRTLGT P-SDDELSFI SAPKARAYIK 269
XP_013902829 LLLS-CPDYT AAIDVWSVGC ILAELLGRKP LFPGKDYVHQ LNLITKAIGS P-GEEDIQFV SSEKARRYLR 228
XP_002955338 LLLS-CSGYT TAIDVWSVGC IFAELLGRKP LFPGKDYVHQ LSLITKIIGS P-SEGELGFI TSEKAKRYIR 303
KXZ46360 LLLS-CSGYT TAIDVWSVGC IFAELLGRKP LFPGKDYVHQ LSLITKIIGS P-TEEELGFI TSEKAKRYIR 299
XP_001700291 LLLS-CSGYT TAIDVWSVGC IFAELLGRKP LFPGKDYVHQ LSLITKVIGS P-SEEELGFI TSEKAKRYIR 298
XP_002503699 LLLS-CSEYT SAIDVWSVGC IFAELLGRKP LFPGKDYVHQ LNLIARVIGS P-SEEEMEFI TSDKARRYIR 293
XP_003059282 LLLS-CAEYT AAIDVWSVGC IFAELLGRKP LFPGKDYVHQ LNLITRVIGS P-SESEMAFI SSDKARRYIR 296
Consensus LLLS-CTKYT XAIDXWSVGC IFAELLGGKP LFPGKDYVHQ LELITDLLGT P-SEEDLAFI RSEKARRYLR
100%
Conservation
0%
4.3bits
Sequence logo
0.0bits

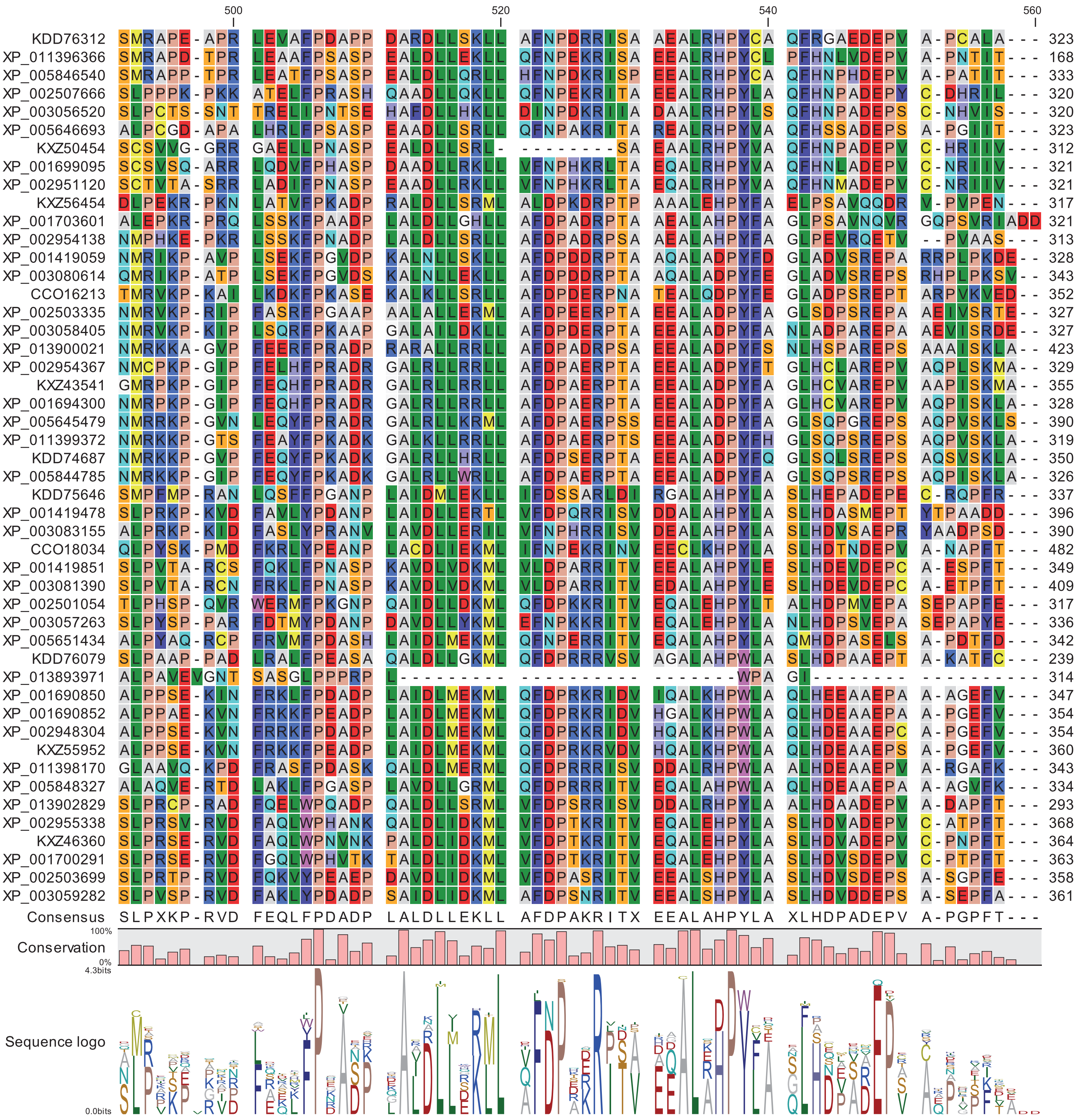
500
520
540
560
KDD76312 323
XP_011396366 168
XP_005846540 333
XP_002507666 320
XP_003056520 320
XP_005646693 323
KXZ50454 312
XP_001699095 321
XP_002951120 321
KXZ56454 317
XP_001703601 321
XP_002954138 313
XP_001419059 328
XP_003080614 343
CCO16213 352
XP_002503335 327
XP_003058405 327
XP_013900021 423
XP_002954367 329
KXZ43541 355
XP_001694300 328
XP_005645479 390
XP_011399372 319
KDD74687 350
XP_005844785 326
KDD75646 337
XP_001419478 396
XP_003083155 390
CCO18034 482
XP_001419851 349
XP_003081390 409
XP_002501054 317
XP_003057263 336
XP_005651434 342
KDD76079 239
XP_013893971 314
XP_001690850 347
XP_001690852 354
XP_002948304 354
KXZ55952 360
XP_011398170 343
XP_005848327 334
XP_013902829 293
XP_002955338 368
KXZ46360 364
XP_001700291 363
XP_002503699 358
XP_003059282 361
Consensus SLPXKP-RVD FEQLFPDADP LALDLLEKLL AFDPAKRITX EEALAHPYLA XLHDPADEPV A-PGPFT---
Conservation
100%
0%
4.3bits
Sequence logo
0.0bits

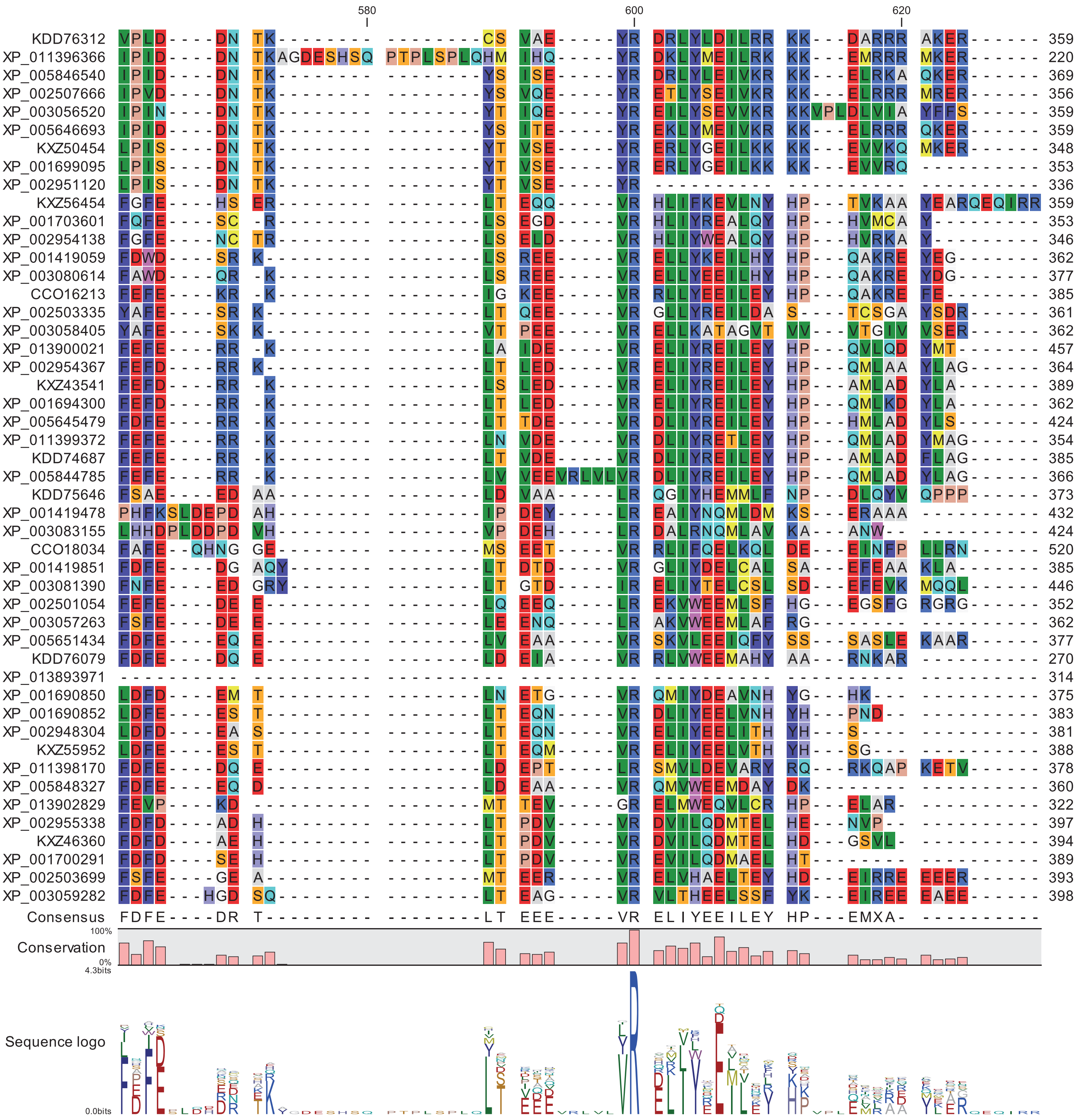
580
600
620
KDD76312 359
XP_011396366 220
XP_005846540 369
XP_002507666 356
XP_003056520 359
XP_005646693 359
KXZ50454 348
XP_001699095 353
XP_002951120 336
KXZ56454 359
XP_001703601 353
XP_002954138 346
XP_001419059 362
XP_003080614 377
CCO16213 385
XP_002503335 361
XP_003058405 362
XP_013900021 457
XP_002954367 364
KXZ43541 389
XP_001694300 362
XP_005645479 424
XP_011399372 354
KDD74687 385
XP_005844785 366
KDD75646 373
XP_001419478 432
XP_003083155 424
CCO18034 520
XP_001419851 385
XP_003081390 446
XP_002501054 352
XP_003057263 362
XP_005651434 377
KDD76079 270
XP_013893971 314
XP_001690850 375
XP_001690852 383
XP_002948304 381
KXZ55952 388
XP_011398170 378
XP_005848327 360
XP_013902829 322
XP_002955338 397
KXZ46360 394
XP_001700291 389
XP_002503699 393
XP_003059282 398
Consensus FDFE----DR T--------- ---------LT EEE-----VR ELIYEEILEY HP---EMXA- ----------
100%
Conservation
0%
4.3bits
Sequence logo
0.0bits

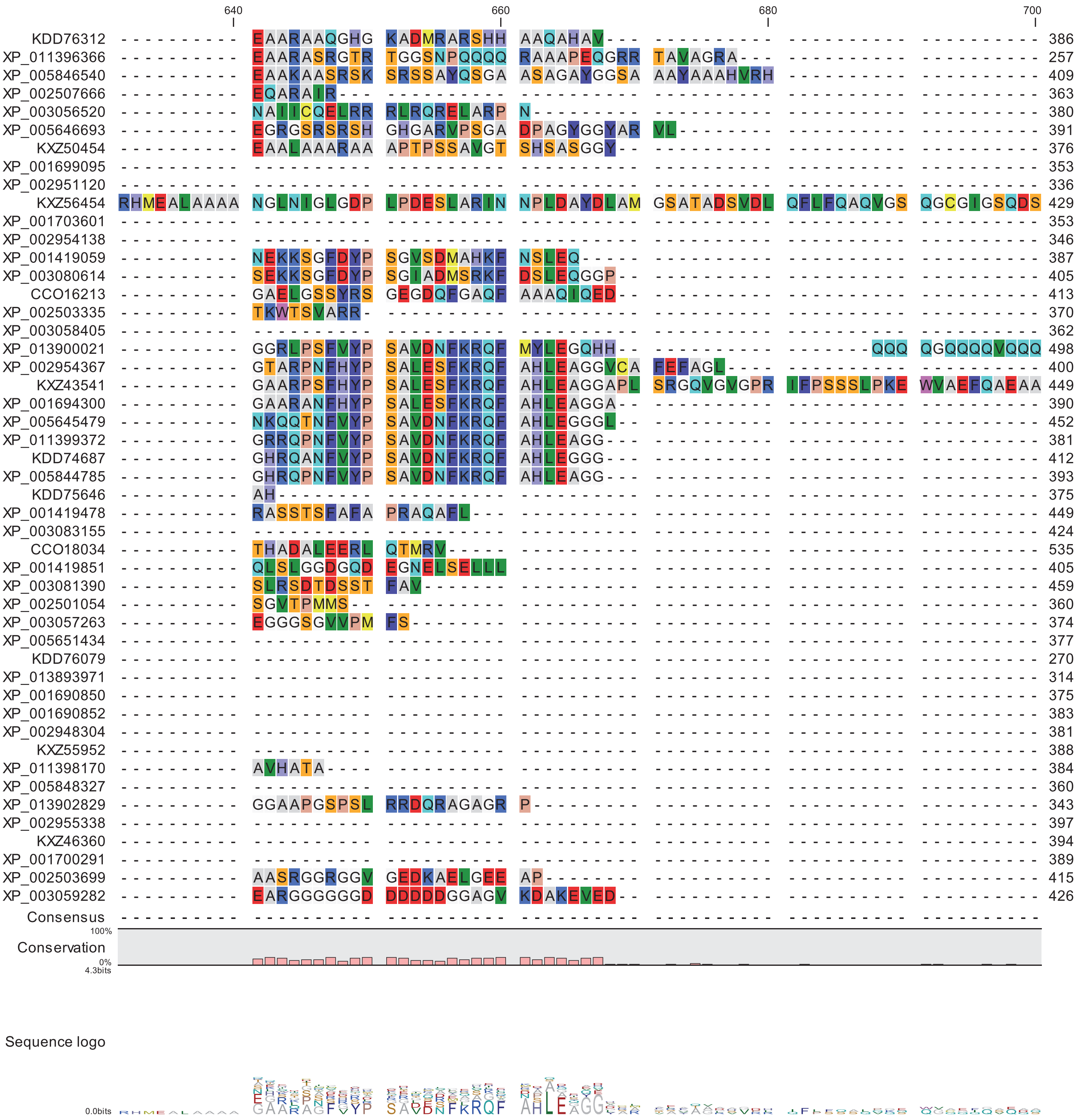
640
660
680
700
KDD76312 ---------- EAARAAQGHG KADMRARSHH AAQAHAV--- ---------- ---------- ---------- 386
XP_011396366 ---------- EAARASRGTR TGGSNPQQQQ RAAAPEQGRR TAVAGRA--- ---------- ---------- 257
XP_005846540 ---------- EAAKAASRSK SRSSAYQSGA ASAGAYGGSA AAYAAAHVRH ---------- ---------- 409
XP_002507666 ---------- EQARAIR--- ---------- ---------- ---------- ---------- ---------- 363
XP_003056520 ---------- NAIICQELRR RLRQRELARP N--------- ---------- ---------- ---------- 380
XP_005646693 ---------- EGRGSRSRSH GHGARVPSGA DPAGYGGYAR VL-------- ---------- ---------- 391
KXZ50454 ---------- EAALAAARAA APTPSSAVGT SHSASGGY-- ---------- ---------- ---------- 376
XP_001699095 ---------- ---------- ---------- ---------- ---------- ---------- ---------- 353
XP_002951120 ---------- ---------- ---------- ---------- ---------- ---------- ---------- 336
KXZ56454 RHMEALAAAA NGLNIGLGDP LPDESLARIN NPLDAYDLAM GSATADSVDL QFLFQAQVGS QGCGIGSQDS 429
XP_001703601 ---------- ---------- ---------- ---------- ---------- ---------- ---------- 353
XP_002954138 ---------- ---------- ---------- ---------- ---------- ---------- ---------- 346
XP_001419059 ---------- NEKKSGFDYP SGVSDMAHKF NSLEQ----- ---------- ---------- ---------- 387
XP_003080614 ---------- SEKKSGFDYP SGIADMSRKF DSLEQGGP-- ---------- ---------- ---------- 405
CCO16213 ---------- GAELGSSYRS GEGDQFGAQF AAAQIQED-- ---------- ---------- ---------- 413
XP_002503335 ---------- TKWTSVARR- ---------- ---------- ---------- ---------- ---------- 370
XP_003058405 ---------- ---------- ---------- ---------- ---------- ---------- ---------- 362
XP_013900021 ---------- GGRLPSFVYP SAVDNFKRQF MYLEGQHH-- ---------- -------QQQ QGQQQQVQQQ 498
XP_002954367 ---------- GTARPNFHYP SALESFKRQF AHLEAGGVCA FEFAGL---- ---------- ---------- 400
KXZ43541 ---------- GAARPSFHYP SALESFKRQF AHLEAGGAPL SRGQVGVGPR IFPSSSLPKE WVAEFQAEAA 449
XP_001694300 ---------- GAARANFHYP SALESFKRQF AHLEAGGA-- ---------- ---------- ---------- 390
XP_005645479 ---------- NKQQTNFVYP SAVDNFKRQF AHLEGGGL-- ---------- ---------- ---------- 452
XP_011399372 ---------- GRRQPNFVYP SAVDNFKRQF AHLEAGG--- ---------- ---------- ---------- 381
KDD74687 ---------- GHRQANFVYP SAVDNFKRQF AHLEGGG--- ---------- ---------- ---------- 412
XP_005844785 ---------- GHRQPNFVYP SAVDNFKRQF AHLEAGG--- ---------- ---------- ---------- 393
KDD75646 ---------- AH-------- ---------- ---------- ---------- ---------- ---------- 375
XP_001419478 ---------- RASSTSFAFA PRAQAFL--- ---------- ---------- ---------- ---------- 449
XP_003083155 ---------- ---------- ---------- ---------- ---------- ---------- ---------- 424
CCO18034 ---------- THADALEERL QTMRV----- ---------- ---------- ---------- ---------- 535
XP_001419851 ---------- QLSLGGDGQD EGNELSELLL ---------- ---------- ---------- ---------- 405
XP_003081390 ---------- SLRSDTDSST FAV------- ---------- ---------- ---------- ---------- 459
XP_002501054 ---------- SGVTPMMS-- ---------- ---------- ---------- ---------- ---------- 360
XP_003057263 ---------- EGGGSGVVPM FS-------- ---------- ---------- ---------- ---------- 374
XP_005651434 ---------- ---------- ---------- ---------- ---------- ---------- ---------- 377
KDD76079 ---------- ---------- ---------- ---------- ---------- ---------- ---------- 270
XP_013893971 ---------- ---------- ---------- ---------- ---------- ---------- ---------- 314
XP_001690850 ---------- ---------- ---------- ---------- ---------- ---------- ---------- 375
XP_001690852 ---------- ---------- ---------- ---------- ---------- ---------- ---------- 383
XP_002948304 ---------- ---------- ---------- ---------- ---------- ---------- ---------- 381
KXZ55952 ---------- ---------- ---------- ---------- ---------- ---------- ---------- 388
XP_011398170 ---------- AVHATA---- ---------- ---------- ---------- ---------- ---------- 384
XP_005848327 ---------- ---------- ---------- ---------- ---------- ---------- ---------- 360
XP_013902829 ---------- GGAAPGSPSL RRDQRAGAGR P--------- ---------- ---------- ---------- 343
XP_002955338 ---------- ---------- ---------- ---------- ---------- ---------- ---------- 397
KXZ46360 ---------- ---------- ---------- ---------- ---------- ---------- ---------- 394
XP_001700291 ---------- ---------- ---------- ---------- ---------- ---------- ---------- 389
XP_002503699 ---------- AASRGGRGGV GEDKAELGEE AP-------- ---------- ---------- ---------- 415
XP_003059282 ---------- EARGGGGGGD DDDDDGGAGV KDAKEVED-- ---------- ---------- ---------- 426
Consensus ---------- ---------- ---------- ---------- ---------- ---------- ----------
100%
Conservation
0%
4.3bits
Sequence logo
0.0bits

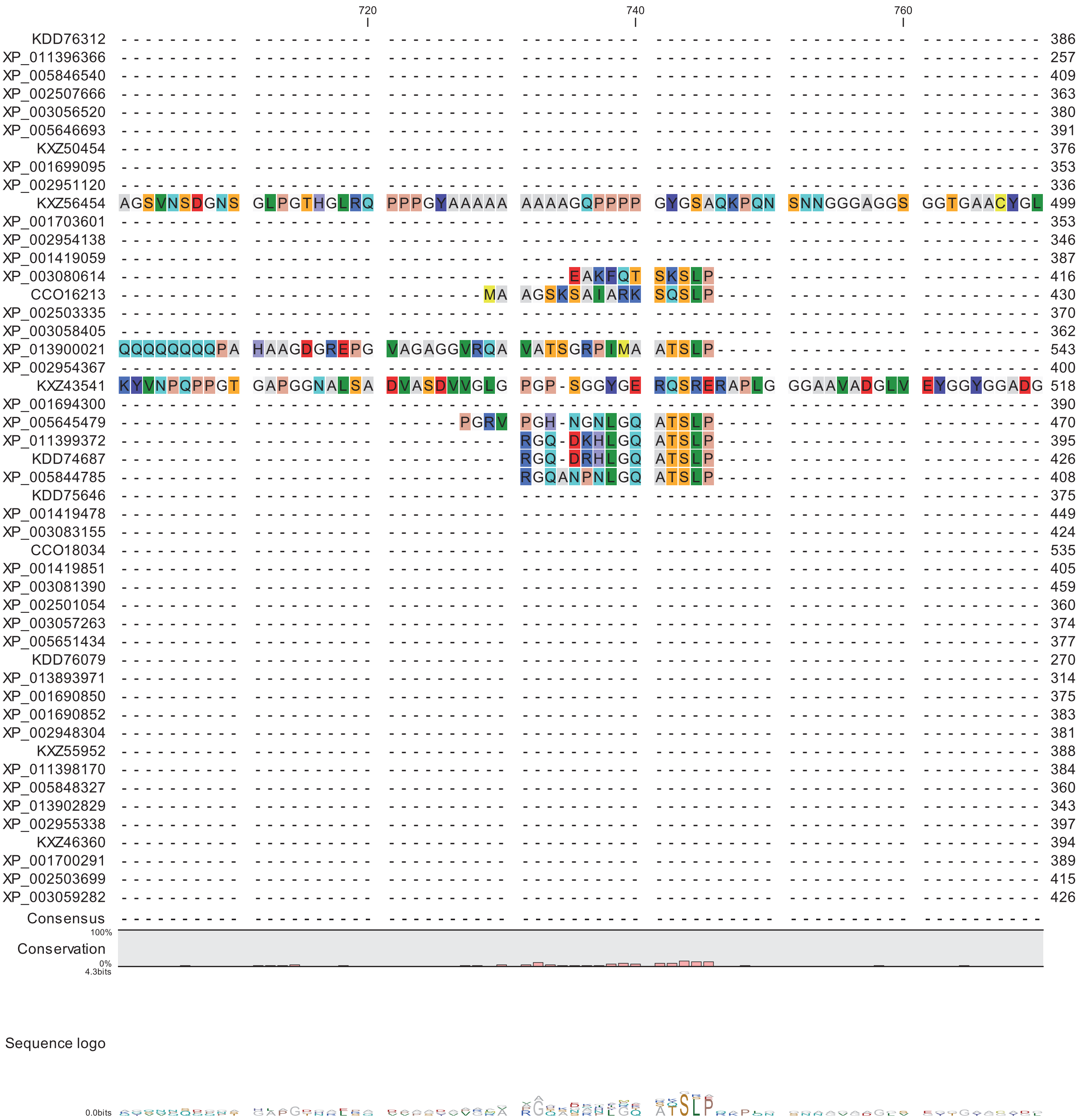
720
740
760
KDD76312 ---------- ---------- ---------- ---------- ---------- ---------- ---------- 386
XP_011396366 ---------- ---------- ---------- ---------- ---------- ---------- ---------- 257
XP_005846540 ---------- ---------- ---------- ---------- ---------- ---------- ---------- 409
XP_002507666 ---------- ---------- ---------- ---------- ---------- ---------- ---------- 363
XP_003056520 ---------- ---------- ---------- ---------- ---------- ---------- ---------- 380
XP_005646693 ---------- ---------- ---------- ---------- ---------- ---------- ---------- 391
KXZ50454 ---------- ---------- ---------- ---------- ---------- ---------- ---------- 376
XP_001699095 ---------- ---------- ---------- ---------- ---------- ---------- ---------- 353
XP_002951120 ---------- ---------- ---------- ---------- ---------- ---------- ---------- 336
KXZ56454 AGSVNSDGNS GLPGTHGLRQ PPPGYAAAAA AAAAGQPPPP GYGSAQKPQN SNNGGGAGGS GGTGAACYGL 499
XP_001703601 ---------- ---------- ---------- ---------- ---------- ---------- ---------- 353
XP_002954138 ---------- ---------- ---------- ---------- ---------- ---------- ---------- 346
XP_001419059 ---------- ---------- ---------- ---------- ---------- ---------- ---------- 387
XP_003080614 ---------- ---------- ---------- ----EAKFQT SKSLP----- ---------- ---------- 416
CCO16213 ---------- ---------- --------MA AGSKSAIARK SQSLP----- ---------- ---------- 430
XP_002503335 ---------- ---------- ---------- ---------- ---------- ---------- ---------- 370
XP_003058405 ---------- ---------- ---------- ---------- ---------- ---------- ---------- 362
XP_013900021 QQQQQQQQPA HAAGDGREPG VAGAGGVRQA VATSGRPIMA ATSLP----- ---------- ---------- 543
XP_002954367 ---------- ---------- ---------- ---------- ---------- ---------- ---------- 400
KXZ43541 KYVNPQPPGT GAPGGNALSA DVASDVVGLG PGP-SGGYGE RQSRERAPLG GGAAVADGLV EYGGYGGADG 518
XP_001694300 ---------- ---------- ---------- ---------- ---------- ---------- ---------- 390
XP_005645479 ---------- ---------- ------PGRV PGH-NGNLGQ ATSLP----- ---------- ---------- 470
XP_011399372 ---------- ---------- ---------- RGQ-DKHLGQ ATSLP----- ---------- ---------- 395
KDD74687 ---------- ---------- ---------- RGQ-DRHLGQ ATSLP----- ---------- ---------- 426
XP_005844785 ---------- ---------- ---------- RGQANPNLGQ ATSLP----- ---------- ---------- 408
KDD75646 ---------- ---------- ---------- ---------- ---------- ---------- ---------- 375
XP_001419478 ---------- ---------- ---------- ---------- ---------- ---------- ---------- 449
XP_003083155 ---------- ---------- ---------- ---------- ---------- ---------- ---------- 424
CCO18034 ---------- ---------- ---------- ---------- ---------- ---------- ---------- 535
XP_001419851 ---------- ---------- ---------- ---------- ---------- ---------- ---------- 405
XP_003081390 ---------- ---------- ---------- ---------- ---------- ---------- ---------- 459
XP_002501054 ---------- ---------- ---------- ---------- ---------- ---------- ---------- 360
XP_003057263 ---------- ---------- ---------- ---------- ---------- ---------- ---------- 374
XP_005651434 ---------- ---------- ---------- ---------- ---------- ---------- ---------- 377
KDD76079 ---------- ---------- ---------- ---------- ---------- ---------- ---------- 270
XP_013893971 ---------- ---------- ---------- ---------- ---------- ---------- ---------- 314
XP_001690850 ---------- ---------- ---------- ---------- ---------- ---------- ---------- 375
XP_001690852 ---------- ---------- ---------- ---------- ---------- ---------- ---------- 383
XP_002948304 ---------- ---------- ---------- ---------- ---------- ---------- ---------- 381
KXZ55952 ---------- ---------- ---------- ---------- ---------- ---------- ---------- 388
XP_011398170 ---------- ---------- ---------- ---------- ---------- ---------- ---------- 384
XP_005848327 ---------- ---------- ---------- ---------- ---------- ---------- ---------- 360
XP_013902829 ---------- ---------- ---------- ---------- ---------- ---------- ---------- 343
XP_002955338 ---------- ---------- ---------- ---------- ---------- ---------- ---------- 397
KXZ46360 ---------- ---------- ---------- ---------- ---------- ---------- ---------- 394
XP_001700291 ---------- ---------- ---------- ---------- ---------- ---------- ---------- 389
XP_002503699 ---------- ---------- ---------- ---------- ---------- ---------- ---------- 415
XP_003059282 ---------- ---------- ---------- ---------- ---------- ---------- ---------- 426
Consensus ---------- ---------- ---------- ---------- ---------- ---------- ----------
100%
Conservation
0%
4.3bits
Sequence logo
0.0bits

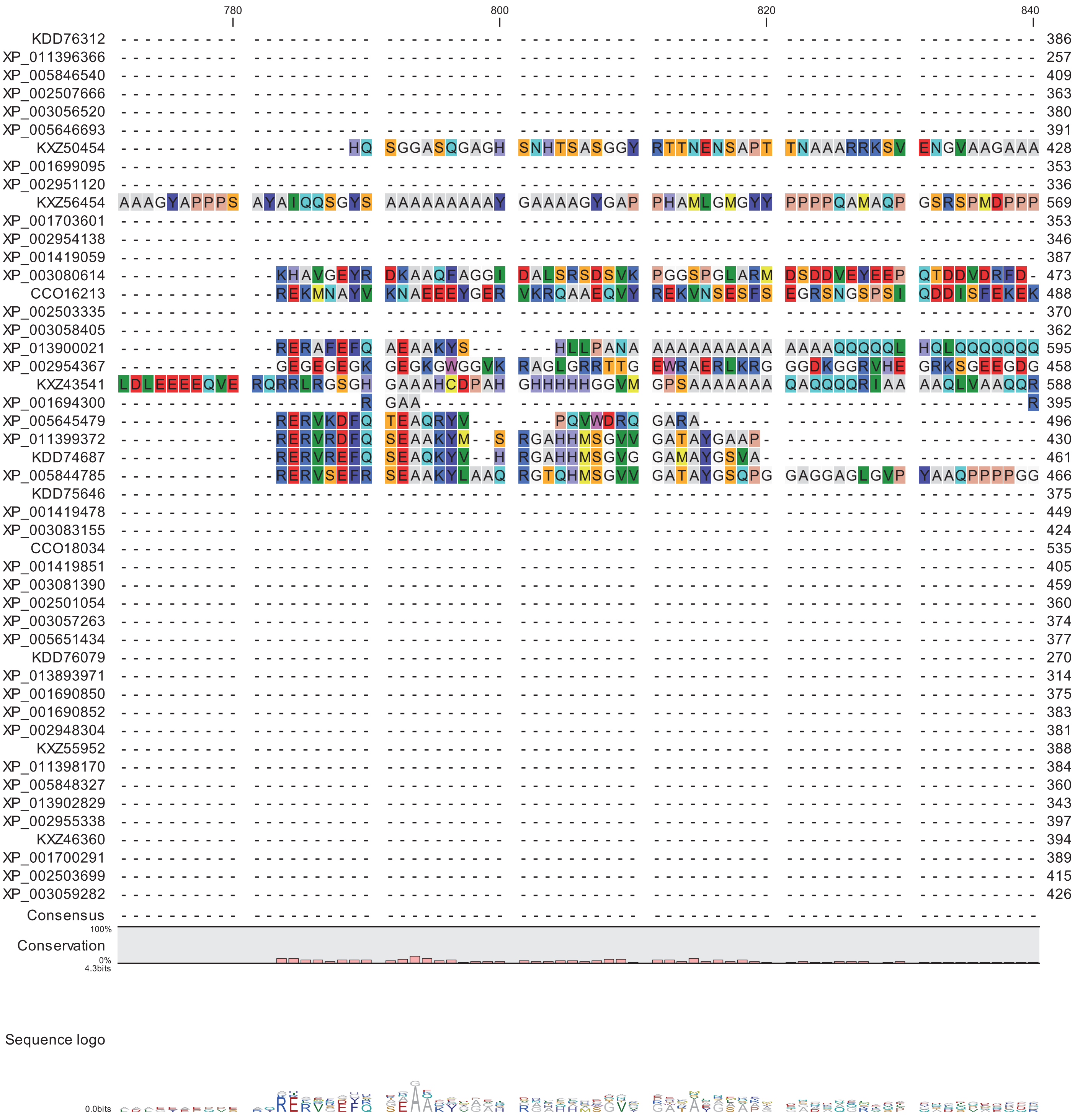
780
800
820
840
KDD76312 386
XP_011396366 257
XP_005846540 409
XP_002507666 363
XP_003056520 380
XP_005646693 391
KXZ50454 --------HQ SGGASQGAGH SNHTSASGGY RTTNENSAPT TNAAARRKSV ENGVAAGAAA 428
XP_001699095 353
XP_002951120 336
KXZ56454 AAAGYAPPPS AYAIQQSGYS AAAAAAAAAY GAAAAGYGAP PHAMLGMGYY PPPPQAMAQP GSRSPMDPPP 569
XP_001703601 353
XP_002954138 346
XP_001419059 387
XP_003080614 --KHAVGEYR DKAAQFAGGI DALSRSDSVK PGGSPGLARM DSDDVEYEEP QTDDVDRFD- 473
CCO16213 --REKMNAYV KNAEEEYGER VKRQAAEQVY REKVNSESFS EGRSNGSPSI QDDISFEKEK 488
XP_002503335 370
XP_003058405 362
XP_013900021 --RERAFEFQ AEAAKYS--- ---HLLPANA AAAAAAAAAA AAAAQQQQQL HQLQQQQQQQ 595
XP_002954367 --GEGEGEGK GEGKGWGGVK RAGLGRRTTG EWRAERLKRG GGDKGGRVHE GRKSGEEGDG 458
KXZ43541 LDLEEEEQVE RQRRLRGSGH GAAAHCDPAH GHHHHHGGVM GPSAAAAAAA QAQQQQRIAA AAQLVAAQQR 588
XP_001694300 ---------R GAA------- ---------R 395
XP_005645479 --RERVKDFQ TEAQRYV--- ---PQVWDRQ GARA------ 496
XP_011399372 --RERVRDFQ SEAAKYM--S RGAHHMSGVV GATAYGAAP- 430
KDD74687 --RERVREFQ SEAQKYV--H RGAHHMSGVG GAMAYGSVA- 461
XP_005844785 --RERVSEFR SEAAKYLAAQ RGTQHMSGVV GATAYGSQPG GAGGAGLGVP YAAQPPPPGG 466
KDD75646 375
XP_001419478 449
XP_003083155 424
CCO18034 535
XP_001419851 405
XP_003081390 459
XP_002501054 360
XP_003057263 374
XP_005651434 377
KDD76079 270
XP_013893971 314
XP_001690850 375
XP_001690852 383
XP_002948304 381
KXZ55952 388
XP_011398170 384
XP_005848327 360
XP_013902829 343
XP_002955338 397
KXZ46360 394
XP_001700291 389
XP_002503699 415
XP_003059282 426
Consensus
100%
Conservation
0%
4.3bits
Sequence logo
0.0bits

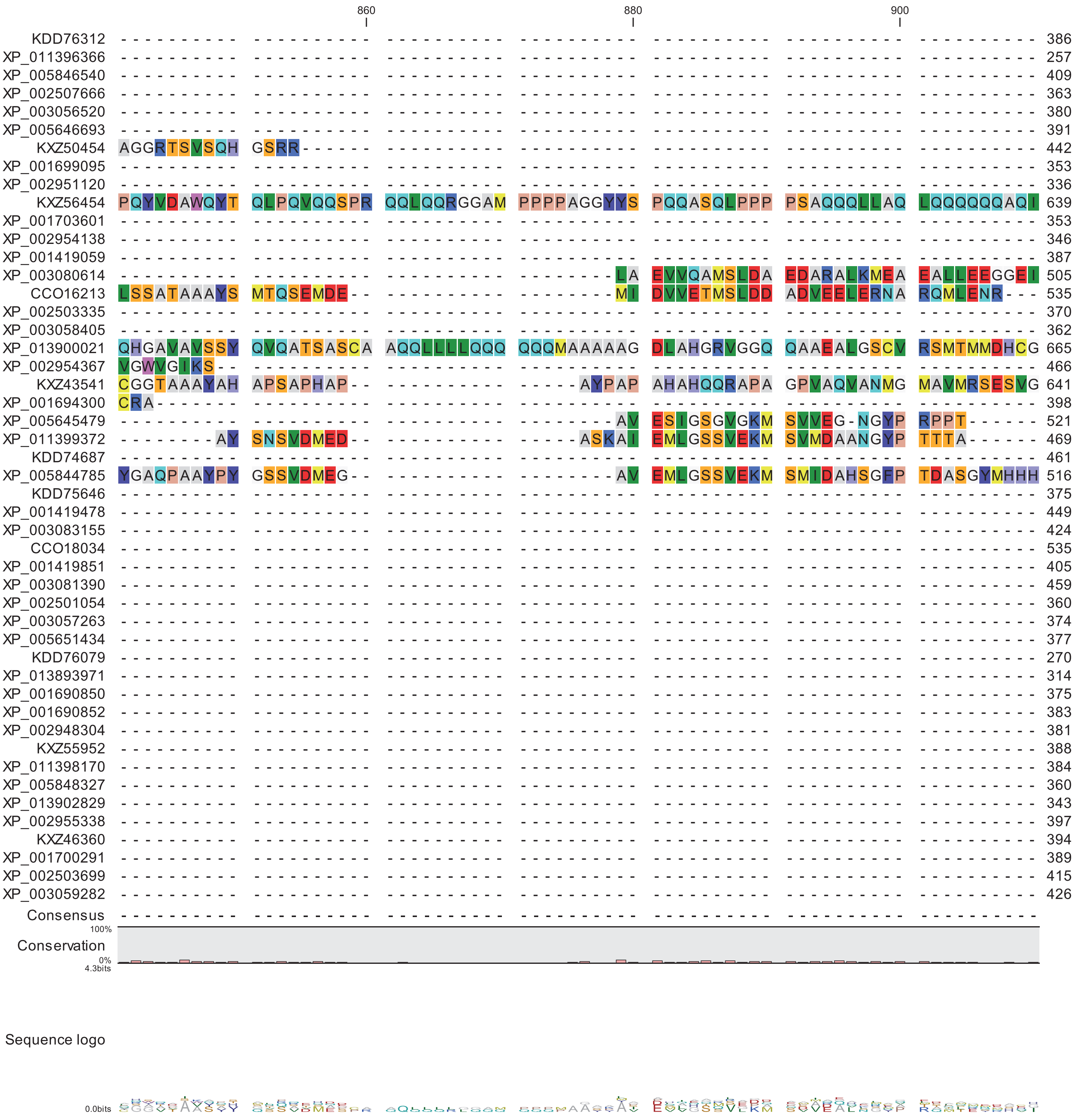

860
880
900
KDD76312 386
XP_011396366 257
XP_005846540 409
XP_002507666 363
XP_003056520 380
XP_005646693 391
KXZ50454 AGGRTSVSQH GSRR 442
XP_001699095 353
XP_002951120 336
KXZ56454 PQYVDAWQYT QLPQVQQSPR QQLQQRGGAM PPPPAGGYYS PQQASQLPPP PSAQQQLLAQ LQQQQQQAQI 639
XP_001703601 353
XP_002954138 346
XP_001419059 387
XP_003080614 LA EVVQAMSLDA EDARALKMEA EALLEEGGEI 505
CCO16213 LSSATAAAYS MTQSEMDE MI DVVETMSLDD ADVEELERNA RQMLENR 535
XP_002503335 370
XP_003058405 362
XP_013900021 QHGAVAVSSY QVQATSASCA AQQLLLLQQQ QQQMAAAAAG DLAHGRVGGQ QAAEALGSCV RSMTMMDHCG 665
XP_002954367 VGWVGIKS 466
KXZ43541 CGGTAAAYAH APSAPHAP AYPAP AHAHQQRAPA GPVAQVANMG MAVMRSESVG 641
XP_001694300 CRA 398
XP_005645479 AV ESIGSGVGKM SVVEG-NGYP RPPT 521
XP_011399372 AY SNSVDMED ASKAI EMLGSSVEKM SVMDAANGYP TTTA 469
KDD74687 461
XP_005844785 YGAQPAAYPY GSSVDMEG AV EMLGSSVEKM SMIDAHSGFP TDASGYMHHH 516
KDD75646 375
XP_001419478 449
XP_003083155 424
CCO18034 535
XP_001419851 405
XP_003081390 459
XP_002501054 360
XP_003057263 374
XP_005651434 377
KDD76079 270
XP_013893971 314
XP_001690850 375
XP_001690852 383
XP_002948304 381
KXZ55952 388
XP_011398170 384
XP_005848327 360
XP_013902829 343
XP_002955338 397
KXZ46360 394
XP_001700291 389
XP_002503699 415
XP_003059282 426
Consensus
100%
Conservation
0%
4.3bits
Sequence logo
0.0bits

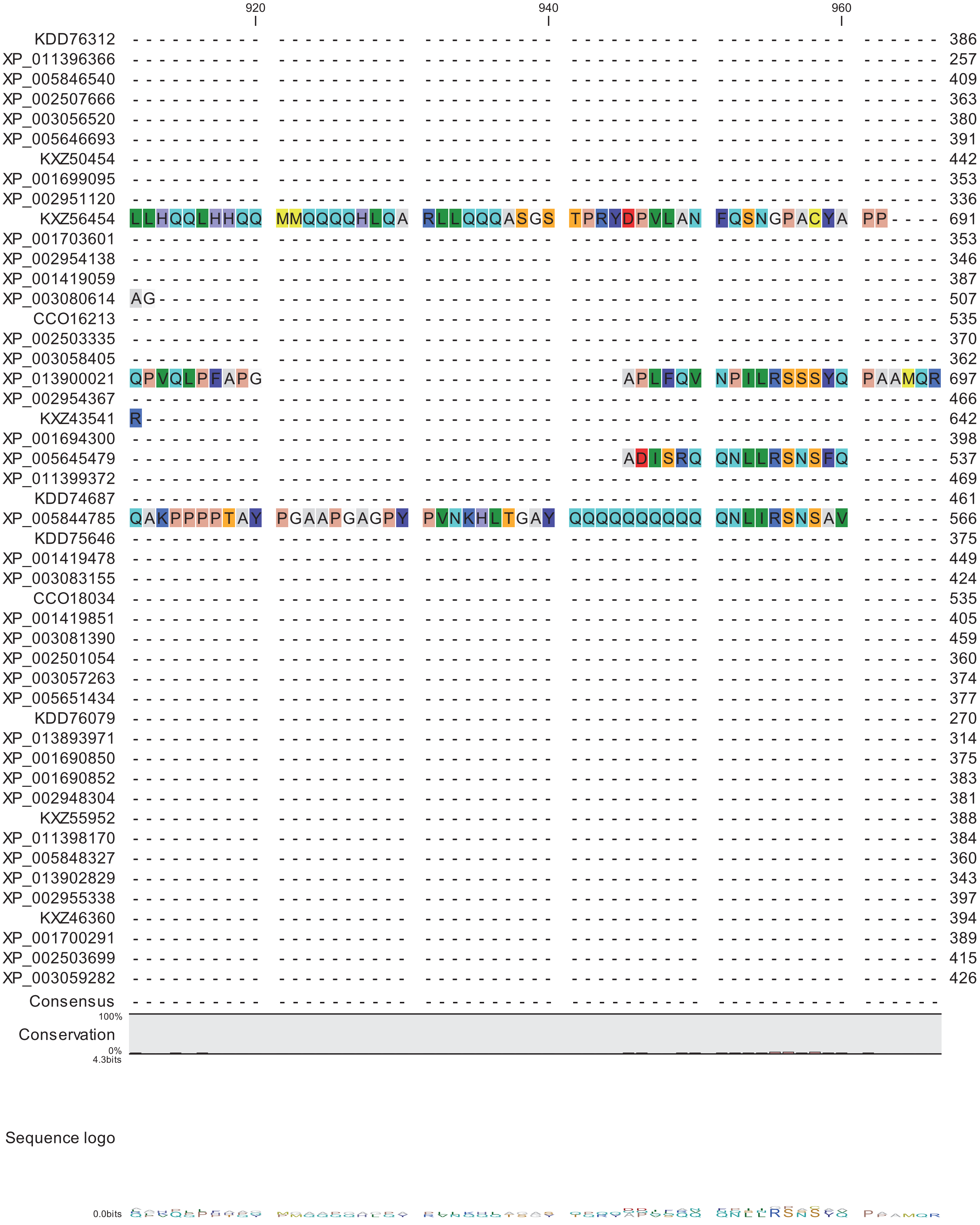

Figure S1

Multiple alignment of MAPK protein sequences from 13 algal species. 48 sequences identified in 13 species (Table 2) were aligned by the MUSCLE alignment method. Amino acids are coloured according to their biochemical properties to visualise conservation and similarities. Consensus and degree of conservation are indicated below each block.

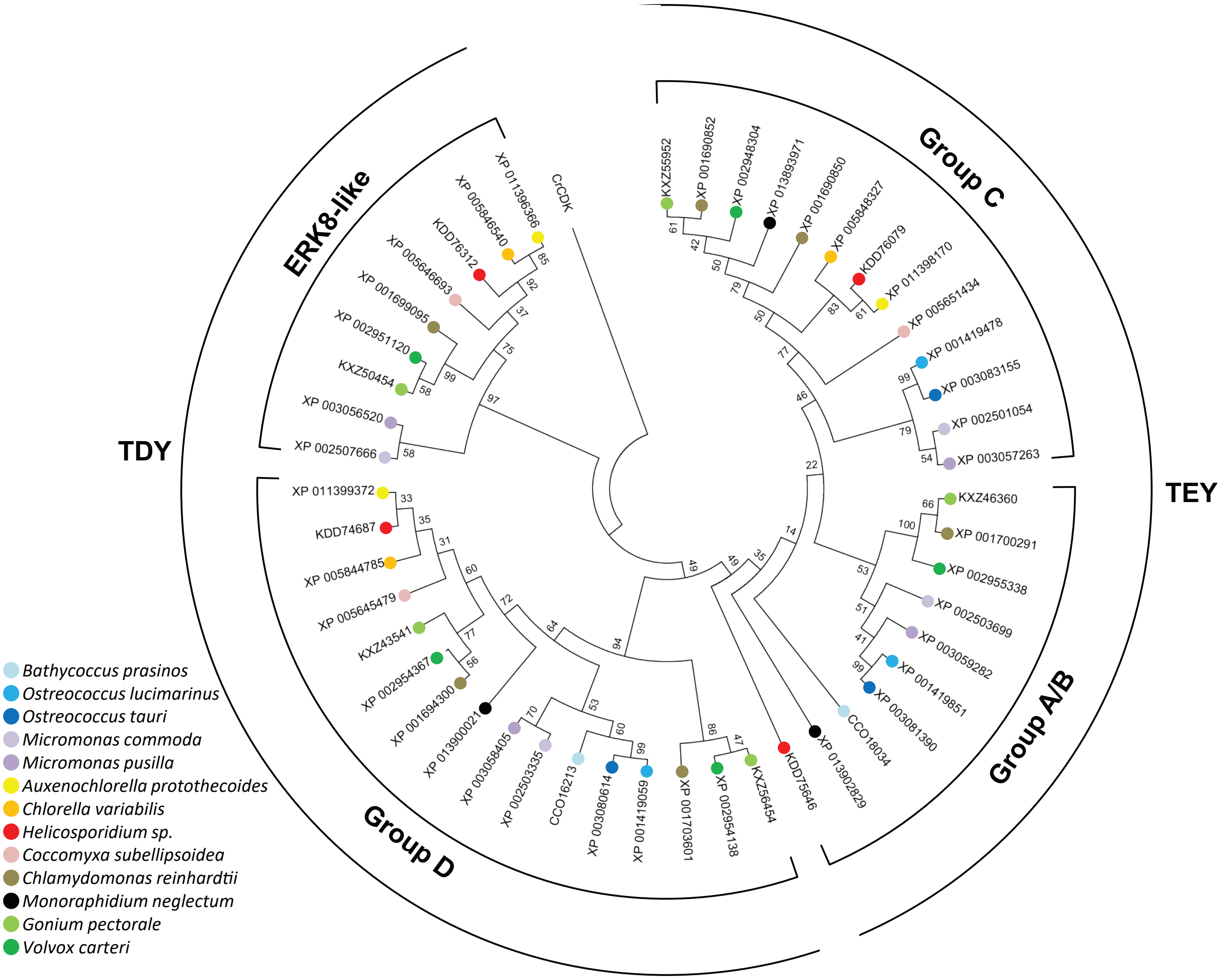

Figure S2

Phylogenetic relationships of MAPK protein sequences in 13 algal species. Full-length amino acid sequences of the 48 identified MAPKs (Table 2) were aligned by the MUSCLE alignment method and the phylogenetic tree was constructed by the Maximum-likelihood method using the MEGA6 software package. A cyclin-dependent kinase (CDKG1) of *Chlamydomonas reinhardtii* was used as outgroup. Species are indicated by the protein IDs in the tree with coloured dots according to the key on the left. Phylogenetic grouping and the type of the conserved phosphorylation site (TxY) are indicated at the inner and outer perimeters, respectively.

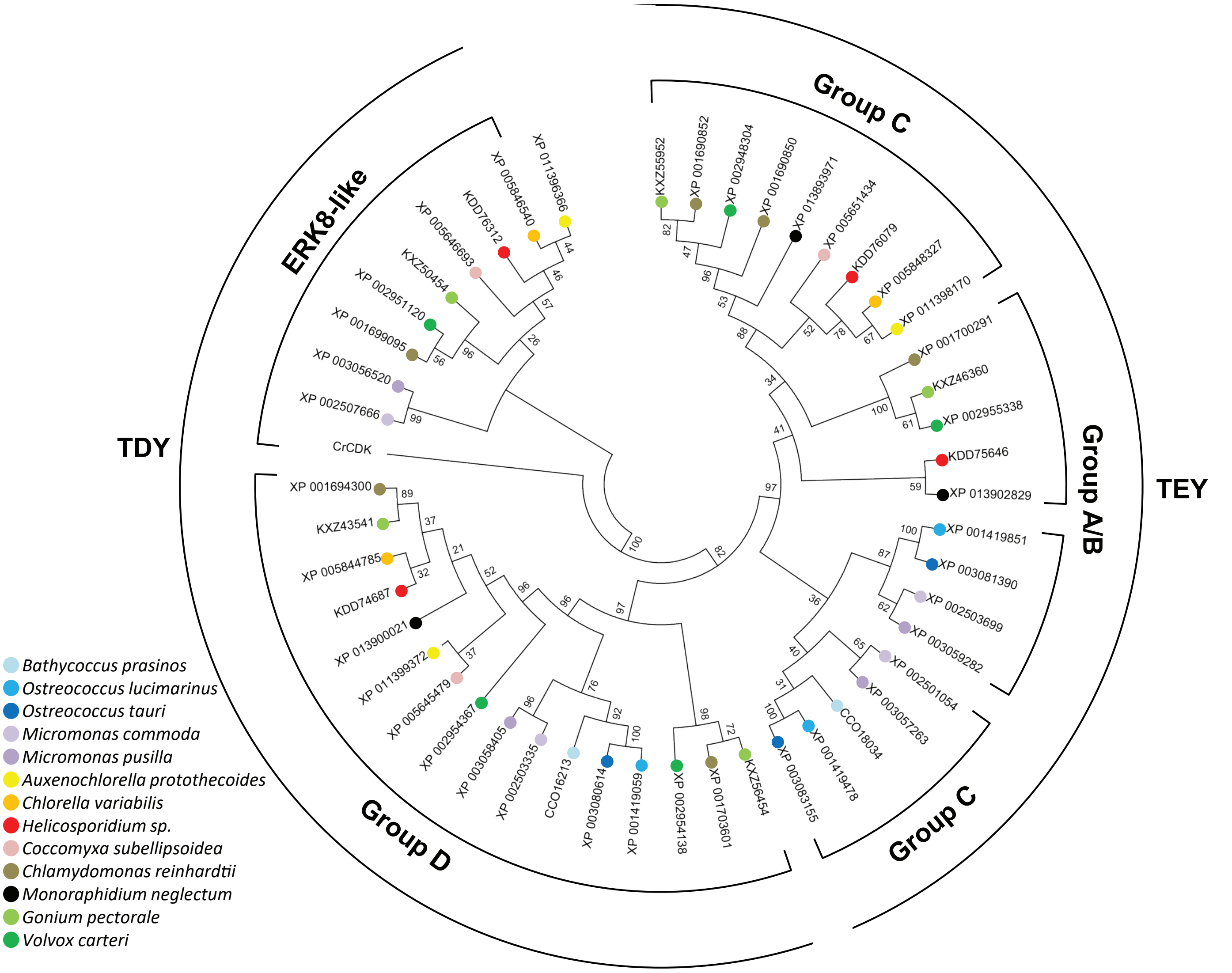

Figure S3

Phylogenetic relationships of MAPK nucleotide sequences in 13 algal species. Full-length nucleotide sequences of the 48 identified MAPKs (Table 2) were aligned by the MUSCLE alignment method and the phylogenetic tree was constructed by the Maximum-likelihood method using the MEGA6 software package. A cyclin-dependent kinase (CDKG1) of *Chlamydomonas reinhardtii* was used as outgroup. Species are indicated by the protein IDs in the tree with coloured dots according to the key on the left. Phylogenetic grouping and the type of the conserved phosphorylation site (TxY) are indicated at the inner and outer perimeters, respectively.

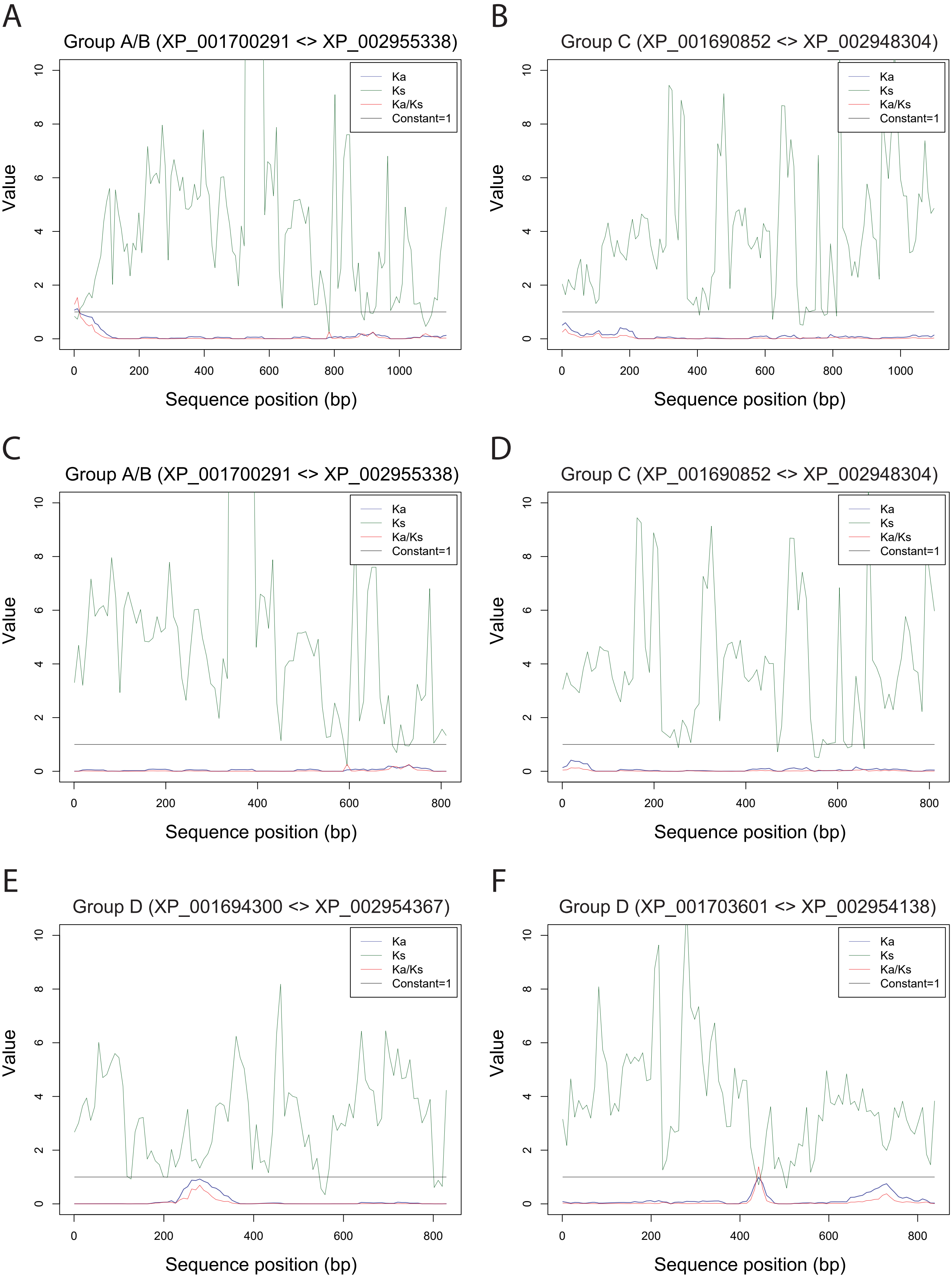

Figure S4

Sliding window analysis of sequence divergence between MAPKs in *C. reinhardtii* and *V. carteri*.

Full-length (A-B) and kinase domain encoding (C-F) nucleotide sequences of the indicated orthologous MAPKs were used at 51-bp window size and 9-bp step size settings for sliding window analysis. The Ka/Ks plots are indicated by red lines.

		ERK8-like									Group A/B										Group C													Group D															
		XP_011396366	XP_001699095	XP_005846540	XP_005646693	KXZ50454	KDD76312	XP_002507666	XP_003056520	XP_002951120	CCO18034	XP_001700291	KXZ46360	KDD75646	XP_002503699	XP_003059282	XP_013902829	XP_001419851	XP_003081390	XP_002955338	XP_011398170	XP_001690850	XP_001690852	XP_005848327	XP_005651434	KXZ55952	KDD76079	XP_002501054	XP_003057263	XP_013893971	XP_001419478	XP_003083155	XP_002948304	XP_011399372	CCO16213	XP_001694300	XP_001703601	XP_005844785	XP_005645479	KXZ43541	KXZ56454	KDD74687	XP_002503335	XP_003058405	XP_013900021	XP_001419059	XP_003080614	XP_002954138	XP_002954367
ERK8-like	XP_011396366																																																
	XP_001699095	0.03																																															
	XP_005846540	0.03	0.02																																														
	XP_005646693	0.05	0.03	0.03																																													
	KXZ50454	0.06	0.02	0.04	0.04																																												
	KDD76312	0.03	0.04	0.03	0.05	0.04																																											
	XP_002507666	0.07	0.05	0.04	0.04	0.05	0.07																																										
	XP_003056520	0.08	0.05	0.04	0.04	0.06	0.07	0.03																																									
	XP_002951120	0.06	0.01	0.03	0.04	0.01	0.05	0.04	0.05																																								
Group A/B	CCO18034	0.18	0.16	0.15	0.15	0.16	0.18	0.16	0.19	0.18																																							
	XP_001700291	0.11	0.11	0.12	0.13	0.11	0.13	0.17	0.18	0.15	0.06																																						
	KXZ46360	0.13	0.13	0.14	0.14	0.11	0.14	0.18	0.20	0.16	0.07	0.01																																					
	KDD75646	0.17	0.16	0.16	0.19	0.16	0.18	0.25	0.25	0.22	0.09	0.06	0.06																																				
	XP_002503699	0.13	0.12	0.12	0.12	0.13	0.13	0.18	0.19	0.16	0.06	0.04	0.04	0.07																																			
	XP_003059282	0.14	0.11	0.13	0.13	0.10	0.12	0.18	0.19	0.13	0.07	0.04	0.04	0.07	0.03																																		
	XP_013902829	0.12	0.14	0.15	0.18	0.14	0.13	0.22	0.25	0.21	0.08	0.03	0.04	0.07	0.05	0.05																																	
	XP_001419851	0.20	0.14	0.15	0.17	0.16	0.19	0.23	0.22	0.17	0.07	0.05	0.06	0.10	0.05	0.05	0.07																																
	XP_003081390	0.18	0.15	0.18	0.16	0.16	0.23	0.21	0.20	0.18	0.10	0.06	0.07	0.09	0.05	0.06	0.07	0.02																															
	XP_002955338	0.15	0.14	0.15	0.15	0.12	0.14	0.18	0.18	0.17	0.08	0.01	0.01	0.08	0.05	0.05	0.04	0.06	0.07																														
Group C	XP_011398170	0.12	0.11	0.09	0.12	0.12	0.12	0.19	0.19	0.15	0.08	0.05	0.06	0.07	0.06	0.05	0.08	0.08	0.08	0.07																													
	XP_001690850	0.12	0.10	0.09	0.11	0.11	0.11	0.16	0.15	0.13	0.06	0.05	0.06	0.07	0.05	0.04	0.06	0.07	0.07	0.07	0.03																												
	XP_001690852	0.12	0.13	0.12	0.12	0.12	0.14	0.18	0.16	0.15	0.07	0.07	0.07	0.08	0.06	0.06	0.06	0.07	0.08	0.08	0.04	0.09																											
	XP_005848327	0.13	0.10	0.12	0.11	0.10	0.13	0.17	0.18	0.14	0.07	0.04	0.05	0.07	0.04	0.04	0.06	0.06	0.06	0.05	0.02	0.02	0.03																										
	XP_005651434	0.14	0.13	0.12	0.14	0.13	0.15	0.17	0.17	0.16	0.07	0.06	0.07	0.10	0.06	0.06	0.08	0.08	0.08	0.08	0.05	0.04	0.04	0.03																									
	KXZ55952	0.13	0.11	0.12	0.12	0.12	0.14	0.18	0.17	0.16	0.08	0.06	0.06	0.08	0.06	0.06	0.06	0.08	0.07	0.08	0.03	0.02	0.01	0.02	0.04																								
	KDD76079	0.11	0.11	0.10	0.13	0.14	0.12	0.19	0.19	0.16	0.08	0.05	0.06	0.06	0.05	0.05	0.06	0.06	0.06	0.07	0.03	0.03	0.04	0.03	0.04	0.04																							
	XP_002501054	0.14	0.14	0.11	0.13	0.13	0.16	0.17	0.18	0.16	0.07	0.04	0.05	0.07	0.05	0.05	0.07	0.07	0.07	0.05	0.06	0.04	0.05	0.04	0.05	0.04	0.05																						
	XP_003057263	0.12	0.11	0.10	0.11	0.12	0.12	0.16	0.16	0.14	0.07	0.05	0.06	0.10	0.06	0.05	0.07	0.07	0.07	0.06	0.05	0.05	0.05	0.04	0.05	0.05	0.05	0.02																					
	XP_013893971	0.33	0.11	0.10	0.13	0.09	0.12	0.18	0.17	0.15	0.07	0.05	0.05	0.07	0.05	0.06	0.05	0.06	0.07	0.06	0.03	0.02	0.03	0.02	0.03	0.02	0.03	0.03	0.04																				
	XP_001419478	0.17	0.17	0.16	0.19	0.15	0.18	0.25	0.24	0.19	0.14	0.10	0.10	0.12	0.09	0.11	0.08	0.10	0.13	0.12	0.09	0.11	0.10	0.07	0.09	0.10	0.09	0.07	0.09	0.08																			
	XP_003083155	0.16	0.15	0.14	0.17	0.17	0.15	0.21	0.21	0.19	0.13	0.09	0.09	0.12	0.10	0.09	0.08	0.10	0.12	0.11	0.07	0.09	0.10	0.07	0.10	0.10	0.07	0.07	0.08	0.08	0.04																		
	XP_002948304	0.16	0.15	0.13	0.14	0.15	0.17	0.18	0.17	0.19	0.08	0.07	0.08	0.10	0.07	0.07	0.08	0.08	0.08	0.09	0.04	0.02	0.02	0.03	0.05	0.01	0.05	0.05	0.06	0.03	0.11	0.11																	
Group D	XP_011399372	0.30	0.16	0.20	0.22	0.21	0.17	0.23	0.24	0.21	0.14	0.10	0.11	0.12	0.11	0.13	0.14	0.16	0.15	0.14	0.13	0.13	0.13	0.12	0.15	0.13	0.13	0.11	0.13	0.09	0.20	0.19	0.15																
	CCO16213	0.24	0.18	0.17	0.21	0.24	0.19	0.21	0.23	0.18	0.16	0.13	0.14	0.15	0.14	0.18	0.16	0.20	0.18	0.14	0.16	0.15	0.14	0.13	0.17	0.15	0.14	0.12	0.14	0.14	0.19	0.20	0.16	0.10															
	XP_001694300	0.18	0.16	0.17	0.18	0.15	0.18	0.24	0.27	0.18	0.15	0.07	0.09	0.11	0.10	0.11	0.11	0.15	0.16	0.11	0.13	0.12	0.13	0.10	0.13	0.12	0.13	0.11	0.12	0.10	0.20	0.17	0.14	0.03	0.08														
	XP_001703601	0.17	0.11	0.11	0.15	0.11	0.12	0.20	0.22	0.14	0.13	0.08	0.11	0.09	0.09	0.08	0.12	0.12	0.13	0.11	0.10	0.10	0.10	0.09	0.12	0.10	0.10	0.10	0.11	0.07	0.15	0.16	0.13	0.05	0.08	0.05													
	XP_005844785	0.15	0.12	0.13	0.15	0.15	0.14	0.22	0.23	0.16	0.11	0.07	0.08	0.09	0.08	0.08	0.10	0.12	0.12	0.09	0.10	0.10	0.09	0.09	0.11	0.09	0.11	0.07	0.08	0.06	0.16	0.15	0.11	0.01	0.09	0.02	0.05												
	XP_005645479	0.21	0.14	0.17	0.18	0.21	0.14	0.21	0.22	0.17	0.18	0.09	0.11	0.14	0.12	0.14	0.12	0.14	0.18	0.13	0.14	0.13	0.14	0.11	0.14	0.14	0.12	0.10	0.11	0.12	0.23	0.19	0.15	0.02	0.10	0.03	0.05	0.02											
	KXZ43541	0.21	0.11	0.14	0.15	0.13	0.13	0.20	0.20	0.15	0.13	0.07	0.09	0.09	0.10	0.09	0.10	0.14	0.14	0.11	0.12	0.11	0.11	0.09	0.12	0.12	0.10	0.09	0.11	0.09	0.19	0.17	0.13	0.04	0.13	0.02	0.05	0.04	0.04										
	KXZ56454	0.19	0.13	0.13	0.14	0.15	0.15	0.20	0.23	0.17	0.13	0.07	0.08	0.11	0.08	0.09	0.11	0.12	0.11	0.10	0.09	0.09	0.10	0.08	0.11	0.09	0.09	0.09	0.10	0.06	0.15	0.14	0.13	0.11	0.14	0.07	0.02	0.08	0.09	0.13									
	KDD74687	0.18	0.14	0.15	0.17	0.18	0.17	0.22	0.26	0.18	0.14	0.09	0.11	0.13	0.10	0.13	0.12	0.12	0.13	0.12	0.13	0.13	0.13	0.11	0.15	0.14	0.11	0.10	0.12	0.09	0.21	0.19	0.14	0.01	0.08	0.02	0.05	0.01	0.03	0.02	0.08								
	XP_002503335	0.16	0.11	0.12	0.14	0.12	0.12	0.18	0.21	0.16	0.11	0.06	0.08	0.10	0.07	0.07	0.10	0.11	0.09	0.09	0.10	0.09	0.09	0.07	0.10	0.09	0.09	0.08	0.08	0.07	0.14	0.14	0.11	0.04	0.04	0.03	0.04	0.02	0.03	0.02	0.05	0.03							
	XP_003058405	0.14	0.12	0.13	0.13	0.12	0.13	0.17	0.19	0.15	0.14	0.07	0.09	0.11	0.07	0.08	0.11	0.11	0.11	0.10	0.11	0.09	0.10	0.08	0.12	0.09	0.12	0.09	0.10	0.07	0.16	0.17	0.13	0.05	0.04	0.04	0.06	0.03	0.03	0.03	0.06	0.03	0.02						
	XP_013900021	0.18	0.10	0.12	0.13	0.13	0.13	0.17	0.19	0.14	0.17	0.09	0.09	0.10	0.10	0.11	0.10	0.14	0.17	0.12	0.11	0.11	0.11	0.09	0.12	0.13	0.11	0.09	0.11	0.08	0.18	0.18	0.15	0.04	0.11	0.02	0.04	0.03	0.04	0.05	0.11	0.03	0.02	0.03					
	XP_001419059	0.21	0.13	0.15	0.16	0.15	0.15	0.20	0.24	0.16	0.14	0.09	0.10	0.12	0.11	0.12	0.14	0.17	0.17	0.11	0.13	0.11	0.11	0.10	0.13	0.10	0.13	0.12	0.12	0.09	0.19	0.16	0.12	0.07	0.06	0.06	0.06	0.05	0.06	0.06	0.08	0.05	0.04	0.05	0.05				
	XP_003080614	0.24	0.13	0.18	0.20	0.24	0.15	0.20	0.23	0.16	0.16	0.10	0.11	0.14	0.12	0.13	0.14	0.18	0.17	0.13	0.15	0.13	0.12	0.12	0.13	0.12	0.14	0.12	0.15	0.09	0.18	0.18	0.14	0.10	0.10	0.07	0.07	0.08	0.09	0.09	0.13	0.07	0.05	0.05	0.09	0.05			
	XP_002954138	0.22	0.15	0.17	0.17	0.15	0.16	0.20	0.22	0.19	0.14	0.10	0.11	0.17	0.11	0.11	0.15	0.16	0.16	0.13	0.13	0.12	0.12	0.12	0.14	0.12	0.15	0.12	0.13	0.11	0.20	0.17	0.14	0.07	0.08	0.06	0.02	0.06	0.06	0.05	0.03	0.06	0.06	0.07	0.05	0.08	0.08		
	XP_002954367	0.24	0.16	0.19	0.19	0.22	0.19	0.20	0.24	0.20	0.15	0.09	0.10	0.13	0.13	0.12	0.14	0.17	0.16	0.12	0.14	0.12	0.14	0.11	0.15	0.13	0.14	0.12	0.12	0.10	0.24	0.18	0.14	0.04	0.11	0.02	0.06	0.04	0.04	0.03	0.11	0.04	0.04	0.04	0.04	0.07	0.10	0.07	

Table S1

Ka/Ks values of the 48 full-length MAPK sequences. Ka/Ks values were calculated for all pairwise combinations of the 48 algal MAPK nucleotide sequences by generating 1175 pairwise alignments using the MUSCLE method of the MEGA6 software package. AXT format files were subsequently prepared using a self-made script. Ka/Ks values were calculated with the KaKs_Calculator2.0 software using the Model Average (MA) method. Ka/Ks values below 1.0 indicate purifying selection.

		ERK8-like								
		XP_011396366	XP_001699095	XP_005846540	XP_005646693	KXZ50454	KDD76312	XP_002507666	XP_003056520	XP_002951120
ERK8-like	XP_011396366									
	XP_001699095	0.04								
	XP_005846540	0.01	0.02							
	XP_005646693	0.04	0.03	0.02						
	KXZ50454	0.05	0.02	0.03	0.03					
	KDD76312	0.02	0.03	0.02	0.04	0.03				
	XP_002507666	0.07	0.05	0.04	0.04	0.05	0.06			
	XP_003056520	0.08	0.04	0.03	0.04	0.04	0.06	0.03		
	XP_002951120	0.06	0.01	0.03	0.03	0.01	0.04	0.04	0.04	
Group A/B	CCO18034	0.15	0.14	0.13	0.13	0.16	0.16	0.13	0.15	0.17
	XP_001700291	0.12	0.10	0.11	0.12	0.11	0.11	0.15	0.15	0.13
	KXZ46360	0.12	0.11	0.12	0.12	0.10	0.12	0.15	0.16	0.13
	KDD75646	0.13	0.12	0.14	0.16	0.14	0.15	0.22	0.22	0.19
	XP_002503699	0.12	0.10	0.11	0.12	0.11	0.11	0.16	0.15	0.14
	XP_003059282	0.11	0.10	0.11	0.11	0.08	0.09	0.15	0.16	0.13
	XP_013902829	0.10	0.12	0.13	0.14	0.12	0.12	0.19	0.22	0.18
	XP_001419851	0.13	0.12	0.12	0.14	0.12	0.14	0.18	0.17	0.15
	XP_003081390	0.15	0.14	0.14	0.15	0.14	0.18	0.18	0.17	0.17
	XP_002955338	0.12	0.12	0.13	0.13	0.10	0.12	0.16	0.16	0.15
Group C	XP_011398170	0.09	0.09	0.09	0.11	0.09	0.10	0.15	0.15	0.14
	XP_001690850	0.09	0.09	0.08	0.10	0.10	0.10	0.14	0.15	0.12
	XP_001690852	0.11	0.11	0.11	0.12	0.11	0.12	0.17	0.15	0.13
	XP_005848327	0.11	0.09	0.10	0.11	0.08	0.12	0.16	0.17	0.11
	XP_005651434	0.12	0.11	0.11	0.13	0.12	0.12	0.15	0.15	0.14
	KXZ55952	0.12	0.11	0.11	0.13	0.11	0.12	0.18	0.16	0.14
	KDD76079	0.10	0.09	0.09	0.12	0.13	0.10	0.16	0.17	0.14
	XP_002501054	0.11	0.10	0.09	0.12	0.10	0.12	0.13	0.15	0.13
	XP_003057263	0.09	0.09	0.08	0.10	0.10	0.09	0.13	0.13	0.11
	XP_013893971	0.13	0.07	0.08	0.11	0.07	0.10	0.15	0.14	0.11
	XP_001419478	0.14	0.15	0.14	0.16	0.13	0.14	0.21	0.19	0.17
	XP_003083155	0.11	0.14	0.13	0.14	0.13	0.13	0.18	0.17	0.16
	XP_002948304	0.13	0.13	0.12	0.14	0.14	0.14	0.17	0.16	0.17
Group D	XP_011399372	0.18	0.14	0.14	0.17	0.13	0.14	0.18	0.19	0.17
	CCO16213	0.14	0.15	0.12	0.16	0.15	0.15	0.17	0.19	0.16
	XP_001694300	0.14	0.15	0.15	0.16	0.13	0.15	0.19	0.23	0.17
	XP_001703601	0.12	0.09	0.09	0.11	0.08	0.10	0.17	0.19	0.12
	XP_005844785	0.11	0.10	0.10	0.12	0.10	0.10	0.17	0.18	0.13
	XP_005645479	0.15	0.13	0.14	0.15	0.12	0.13	0.17	0.18	0.15
	KXZ43541	0.14	0.12	0.12	0.13	0.11	0.12	0.16	0.17	0.15
	KXZ56454	0.13	0.12	0.11	0.15	0.10	0.13	0.20	0.19	0.17
	KDD74687	0.14	0.12	0.13	0.15	0.11	0.13	0.19	0.21	0.15
	XP_002503335	0.13	0.10	0.10	0.12	0.10	0.10	0.14	0.17	0.13
	XP_003058405	0.12	0.10	0.11	0.12	0.10	0.11	0.15	0.16	0.13
	XP_013900021	0.14	0.09	0.12	0.12	0.09	0.11	0.15	0.18	0.14
	XP_001419059	0.17	0.11	0.12	0.14	0.12	0.12	0.16	0.18	0.13
	XP_003080614	0.17	0.12	0.12	0.15	0.13	0.13	0.16	0.18	0.15
	XP_002954138	0.18	0.12	0.15	0.14	0.11	0.13	0.17	0.19	0.15
	XP_002954367	0.17	0.14	0.14	0.16	0.14	0.15	0.16	0.19	0.17

		Group A/B									
		CCO18034	XP_001700291	KXZ46360	KDD75646	XP_002503699	XP_003059282	XP_013902829	XP_001419851	XP_003081390	XP_002955338
Group A/B	CCO18034										
	XP_001700291	0.04									
	KXZ46360	0.04	0.01								
	KDD75646	0.07	0.05	0.05							
	XP_002503699	0.03	0.02	0.02	0.04						
	XP_003059282	0.04	0.02	0.02	0.04	0.02					
	XP_013902829	0.06	0.03	0.03	0.05	0.04	0.04				
	XP_001419851	0.05	0.03	0.03	0.05	0.03	0.03	0.05			
	XP_003081390	0.05	0.03	0.04	0.06	0.03	0.03	0.05	0.01		
	XP_002955338	0.05	0.01	0.01	0.06	0.03	0.03	0.04	0.04	0.04	
Group C	XP_011398170	0.05	0.03	0.04	0.05	0.04	0.04	0.05	0.05	0.05	0.04
	XP_001690850	0.04	0.02	0.04	0.05	0.03	0.03	0.05	0.04	0.04	0.03
	XP_001690852	0.05	0.03	0.04	0.05	0.04	0.04	0.04	0.05	0.05	0.04
	XP_005848327	0.05	0.03	0.03	0.05	0.03	0.03	0.05	0.04	0.04	0.03
	XP_005651434	0.05	0.03	0.04	0.06	0.03	0.03	0.05	0.05	0.05	0.04
	KXZ55952	0.05	0.03	0.04	0.05	0.04	0.04	0.04	0.05	0.05	0.04
	KDD76079	0.06	0.03	0.04	0.04	0.04	0.04	0.05	0.05	0.04	0.04
	XP_002501054	0.05	0.03	0.03	0.06	0.03	0.03	0.05	0.04	0.05	0.03
	XP_003057263	0.05	0.03	0.04	0.07	0.04	0.04	0.06	0.05	0.05	0.04
	XP_013893971	0.04	0.02	0.02	0.04	0.02	0.03	0.03	0.03	0.03	0.03
	XP_001419478	0.08	0.06	0.07	0.09	0.06	0.06	0.06	0.07	0.07	0.07
	XP_003083155	0.08	0.05	0.06	0.08	0.05	0.06	0.06	0.07	0.07	0.07
	XP_002948304	0.05	0.04	0.04	0.06	0.04	0.05	0.06	0.05	0.06	0.05
Group D	XP_011399372	0.11	0.07	0.08	0.10	0.08	0.08	0.11	0.11	0.11	0.10
	CCO16213	0.12	0.09	0.10	0.11	0.10	0.11	0.12	0.13	0.13	0.11
	XP_001694300	0.12	0.06	0.07	0.10	0.07	0.08	0.10	0.10	0.12	0.09
	XP_001703601	0.11	0.07	0.07	0.09	0.07	0.07	0.10	0.09	0.10	0.08
	XP_005844785	0.09	0.04	0.05	0.07	0.05	0.06	0.08	0.08	0.09	0.06
	XP_005645479	0.11	0.06	0.07	0.09	0.08	0.08	0.10	0.10	0.11	0.09
	KXZ43541	0.10	0.05	0.06	0.08	0.06	0.06	0.09	0.08	0.09	0.07
	KXZ56454	0.11	0.06	0.08	0.10	0.06	0.08	0.10	0.09	0.10	0.08
	KDD74687	0.11	0.06	0.06	0.09	0.07	0.07	0.09	0.09	0.10	0.07
	XP_002503335	0.10	0.04	0.06	0.07	0.05	0.05	0.09	0.07	0.08	0.07
	XP_003058405	0.11	0.06	0.08	0.08	0.06	0.06	0.11	0.08	0.09	0.09
	XP_013900021	0.10	0.05	0.05	0.08	0.06	0.06	0.08	0.08	0.09	0.07
	XP_001419059	0.12	0.07	0.07	0.10	0.08	0.08	0.12	0.11	0.13	0.08
	XP_003080614	0.12	0.08	0.08	0.10	0.09	0.09	0.12	0.11	0.13	0.10
	XP_002954138	0.13	0.08	0.09	0.14	0.09	0.10	0.13	0.12	0.13	0.11
	XP_002954367	0.12	0.07	0.08	0.11	0.08	0.08	0.11	0.12	0.13	0.09

		Group C												
		XP_011398170	XP_001690850	XP_001690852	XP_005848327	XP_005651434	KXZ55952	KDD76079	XP_002501054	XP_003057263	XP_013893971	XP_001419478	XP_003083155	XP_002948304
Group C	XP_011398170													
	XP_001690850	0.02												
	XP_001690852	0.02	0.06											
	XP_005848327	0.01	0.02	0.02										
	XP_005651434	0.03	0.03	0.03	0.02									
	KXZ55952	0.02	0.01	0.00	0.02	0.02								
	KDD76079	0.04	0.02	0.03	0.02	0.03	0.03							
	XP_002501054	0.04	0.03	0.03	0.03	0.04	0.03	0.04						
	XP_003057263	0.03	0.03	0.04	0.03	0.04	0.03	0.04	0.02					
	XP_013893971	0.02	0.02	0.01	0.01	0.02	0.01	0.03	0.03	0.03				
	XP_001419478	0.06	0.06	0.06	0.05	0.06	0.06	0.06	0.05	0.05	0.05			
	XP_003083155	0.05	0.05	0.06	0.05	0.06	0.06	0.05	0.05	0.06	0.04	0.05		
	XP_002948304	0.03	0.01	0.01	0.02	0.03	0.01	0.04	0.04	0.04	0.01	0.07	0.07	
Group D	XP_011399372	0.10	0.10	0.11	0.09	0.13	0.11	0.11	0.09	0.09	0.07	0.17	0.17	0.13
	CCO16213	0.11	0.11	0.12	0.10	0.13	0.11	0.12	0.10	0.11	0.10	0.15	0.14	0.12
	XP_001694300	0.10	0.10	0.11	0.09	0.10	0.10	0.11	0.08	0.08	0.07	0.17	0.15	0.12
	XP_001703601	0.08	0.08	0.09	0.07	0.09	0.09	0.08	0.08	0.08	0.06	0.13	0.12	0.11
	XP_005844785	0.07	0.07	0.07	0.06	0.08	0.07	0.08	0.06	0.07	0.05	0.12	0.12	0.09
	XP_005645479	0.10	0.09	0.09	0.08	0.11	0.09	0.10	0.08	0.09	0.08	0.16	0.13	0.10
	KXZ43541	0.08	0.07	0.08	0.07	0.09	0.08	0.08	0.07	0.07	0.06	0.13	0.12	0.09
	KXZ56454	0.08	0.08	0.09	0.07	0.10	0.08	0.08	0.08	0.08	0.06	0.13	0.12	0.11
	KDD74687	0.09	0.09	0.09	0.08	0.11	0.09	0.09	0.07	0.08	0.06	0.14	0.14	0.10
	XP_002503335	0.07	0.07	0.07	0.06	0.08	0.07	0.08	0.06	0.06	0.05	0.12	0.11	0.09
	XP_003058405	0.09	0.08	0.09	0.07	0.09	0.08	0.10	0.07	0.11	0.06	0.14	0.12	0.11
	XP_013900021	0.08	0.08	0.08	0.07	0.09	0.08	0.10	0.07	0.07	0.06	0.13	0.12	0.09
	XP_001419059	0.09	0.09	0.10	0.08	0.10	0.09	0.10	0.08	0.09	0.07	0.14	0.13	0.10
	XP_003080614	0.10	0.09	0.10	0.09	0.10	0.10	0.11	0.09	0.09	0.07	0.15	0.13	0.11
	XP_002954138	0.12	0.11	0.11	0.10	0.11	0.11	0.13	0.10	0.11	0.09	0.17	0.15	0.13
	XP_002954367	0.10	0.10	0.10	0.09	0.11	0.10	0.12	0.09	0.09	0.08	0.17	0.16	0.11

		Group D															
		XP_011399372	CCO16213	XP_001694300	XP_001703601	XP_005844785	XP_005645479	KXZ43541	KXZ56454	KDD74687	XP_002503335	XP_003058405	XP_013900021	XP_001419059	XP_003080614	XP_002954138	XP_002954367
Group D	XP_011399372																
	CCO16213	0.04															
	XP_001694300	0.02	0.05														
	XP_001703601	0.04	0.07	0.04													
	XP_005844785	0.01	0.03	0.01	0.10												
	XP_005645479	0.01	0.04	0.02	0.05	0.01											
	KXZ43541	0.01	0.04	0.01	0.04	0.01	0.01										
	KXZ56454	0.05	0.06	0.05	0.02	0.04	0.04	0.04									
	KDD74687	0.01	0.03	0.02	0.04	0.01	0.01	0.01	0.04								
	XP_002503335	0.02	0.02	0.02	0.03	0.01	0.02	0.01	0.03	0.02							
	XP_003058405	0.03	0.03	0.03	0.04	0.02	0.02	0.02	0.04	0.02	0.01						
	XP_013900021	0.02	0.04	0.02	0.03	0.02	0.02	0.01	0.04	0.02	0.01	0.02					
	XP_001419059	0.04	0.04	0.04	0.05	0.03	0.04	0.03	0.06	0.03	0.03	0.03	0.03				
	XP_003080614	0.04	0.04	0.05	0.05	0.03	0.04	0.04	0.06	0.04	0.03	0.03	0.04	0.03			
	XP_002954138	0.06	0.07	0.05	0.02	0.04	0.05	0.04	0.02	0.05	0.04	0.05	0.04	0.06	0.07		
	XP_002954367	0.02	0.04	0.01	0.05	0.01	0.01	0.01	0.06	0.01	0.02	0.03	0.02	0.04	0.05	0.05	

Table S2

Ka/Ks values of the 48 MAPK kinase domain sequences. Ka/Ks values were calculated for all pairwise combinations of the 48 algal MAPK kinase domain encoding nucleotide sequences by generating 1175 pairwise alignments using the MUSCLE method of the MEGA6 software package. AXT format files were subsequently prepared using a self-made script. Ka/Ks values were calculated with the KaKs_Calculator2.0 software using the Model Average (MA) method. Ka/Ks values below 1.0 indicate purifying selection.
